# A new approach to grain boundary engineering for nanocrystalline materials

**DOI:** 10.3762/bjnano.7.176

**Published:** 2016-11-25

**Authors:** Shigeaki Kobayashi, Sadahiro Tsurekawa, Tadao Watanabe

**Affiliations:** 1 Division of Mechanical Engineering, Department of Innovative Engineering, Faculty of Engineering, Ashikaga Institute of Technology, Omae 268-1, Ashikaga, Tochigi 326-8558, Japan; 2 Department of Materials Science and Engineering, Graduate School of Science and Technology, Kumamoto University, Kumamoto 860-8555, Japan,; 3 Key Laboratory for Anisotropy and Texture of Materials, Northeastern University, Shenyang 110004, China, Formerly, Tohoku University, Sendai, Japan

**Keywords:** electrical resistivity control, fractal analysis, grain boundary engineering (GBE), intergranular fracture control, nanocrystalline materials

## Abstract

A new approach to grain boundary engineering (GBE) for high performance nanocrystalline materials, especially those produced by electrodeposition and sputtering, is discussed on the basis of some important findings from recently available results on GBE for nanocrystalline materials. In order to optimize their utility, the beneficial effects of grain boundary microstructures have been seriously considered according to the almost established approach to GBE. This approach has been increasingly recognized for the development of high performance nanocrystalline materials with an extremely high density of grain boundaries and triple junctions. The effectiveness of precisely controlled grain boundary microstructures (quantitatively characterized by the grain boundary character distribution (GBCD) and grain boundary connectivity associated with triple junctions) has been revealed for recent achievements in the enhancement of grain boundary strengthening, hardness, and the control of segregation-induced intergranular brittleness and intergranular fatigue fracture in electrodeposited nickel and nickel alloys with initial submicrometer-grained structure. A new approach to GBE based on fractal analysis of grain boundary connectivity is proposed to produce high performance nanocrystalline or submicrometer-grained materials with desirable mechanical properties such as enhanced fracture resistance. Finally, the potential power of GBE is demonstrated for high performance functional materials like gold thin films through precise control of electrical resistance based on the fractal analysis of the grain boundary microstructure.

## Review

### Introduction

Nanocrystalline metals and alloys have been receiving increased interest from many researchers because of their unique mechanical [1–20] and functional [21–23] properties, since Birringer, Herr and Gleiter first reported on the processing of nanocrystalline materials and the important characterization of their unique properties in 1986 [1]. Nanocrystalline or nanostructured crystalline materials have opened a new horizon toward the generation of enhanced strength beyond the expectation from the Hall–Petch relationship for conventional polycrystalline structural materials with ordinary grain structure, and conventional grain size range. It is evident that the much higher strength of nanocrystalline materials compared to ordinary polycrystals originates from the extensive interaction between grain boundaries and dislocations. On the other hand, poor ductility and severe brittleness of nanocrystalline materials have been generally observed and still remain unsolved, even beyond our control based on the current discipline of materials science and engineering (MSE).

The unique bulk properties of existing nanocrystalline materials are known to be ascribed to the presence of extremely high density grain boundaries and triple junctions. This is often associated with the nonequilibrium deformation of microstructures introduced by severe plastic deformation (SPD) with less thermal stability, excess structural defects and chemical composition by segregation to grain boundaries and interfaces [12,15,24–29]. Since the concept of grain boundary design and control was first proposed by Watanabe [30], an increasing number of researchers have been involved in the development of high performance polycrystalline materials, including nanocrystalline or nanostructured materials. Engineering applications were successfully achieved first by Aust, Palumbo, Erb and their coworkers [31–32] and then by the authors of this work [33–35]. The nonequilibrium structure, structural defect and chemical composition by segregation as well as grain boundary character as the importance of grain boundary segregation have already been discussed for polycrystalline materials [30,36]. Watanabe et al. [25] have reviewed the recent achievements in GBE by magnetic field application for powerful control of abnormal grain growth and intergranular brittleness due to segregation of detrimental elements in nanocrystalline and ordinary polycrystalline materials [24–25]. More recently, Raabe et al. [27–28] proposed grain boundary segregation engineering for improvement of material properties, such as the stabilization of grains in nanocrystalline steel by carbon and solute element segregation. Kalidindi et al. [29] have suggested that the stability of the nanocrystalline structure is improved by preferential segregation of solute atoms to grain boundaries because their excess free energy can be reduced. Therefore, it is very likely that the grain boundary microstructure characterized by appropriate microstructural parameters (e.g., grain boundary character distribution (GBCD) [30], grain boundary connectivity [30] and triple junction character distribution (TJCD)) may dominantly affect and control the bulk mechanical, physicochemical, electro-magnetic and other grain-boundary-related properties in nanocrystalline materials, as well as ordinary polycrystalline materials.

Accordingly, it is reasonable to consider that the GBE approach that the authors of this work have been deeply involved so far should become of increasing importance in the development of high performance and multifunctional nanocrystalline materials. In recent years, the control of brittle fracture [37–38], creep deformation [39–41], fatigue fracture [42–45], corrosion [46–49] and stress corrosion cracking [40–41,50–51] have been successfully achieved by applying the concept of GBE based on the control of GBCD and grain boundary connectivity in polycrystalline engineering materials. GBE has been extensively achieved by the incorporation of a high fraction of Σ3^n^ boundaries by annealing in low-stacking fault energy FCC metals and alloys. However, it should be noted that the utility of GBE for control of brittleness was already demonstrated for BCC materials with high-stacking fault energy, such as in the very early work of the present author on Fe–6.5 mass % Si alloy with excellent soft magnetic properties but severe brittleness. Ductile, high performance Fe–6.5 mass % Si ribbon material was successfully produced by precise control of grain boundary microstructure associated with the evolution of a sharp <110> texture in the 1980s [52], soon after the first report on nanocrystalline materials.

A number of excellent review papers have been published on nanocrystalline materials produced by different processing methods up to now. The importance of the dominant occurrence of the extremely high density of grain boundaries (in single- phase materials) and interphase boundaries (in multiphase materials such as steels and composite alloys) is generally well understood with respect to the generation of the unique bulk properties of nanocrystalline materials. It is suggested that the dominant effects of grain boundaries on bulk properties of nanocrystalline structural or functional materials should be examined in relation to the applied processing methods, because the details of generation mechanisms of grain boundaries are strongly affected by processing routes and conditions [53–54]. As for those bulk ultrafine-grained (UFG) materials produced by severe plastic deformation (SPD) (e.g., equal-channel angular pressing (ECAP) and high-pressure torsion (HPT) initiated by Valiev and Langdon [55–56]), and also by other quite different methods such as crystallization from amorphous solids [57], extensive work has been performed on the mechanical properties of bulk UFG and nanostructured materials and excellent reviews have been written. Among those previous reviews [19–20,55–61], the most recent review by Pineau et al. [20] may help the reader to understand the past, present and future prospect of this research area, especially on fracture and fatigue of nanostructured metallic materials.

On the other hand, in the present article, a new approach to GBE is introduced for enhanced strength and brittle fracture control in structural and functional nanocrystalline materials produced by electrodeposition and sputtering. This was first applied by Gleiter and coworkers during the very early stage development of nanocrystalline materials [1–2] and was later practically applied by Palumbo, Erb and Aust for the development of high performance structural engineering materials [3,62–63]. For this objective, first, we will discuss the effect of grain boundary microstructures, characterized by the grain boundary character and triple junction character, on the bulk mechanical properties such as hardness and on control of segregation-induced intergranular brittleness and fatigue fracture. Then we will introduce a new approach to GBE based on fractal analysis of grain boundary microstructure for development of nanocrystalline materials with high performance and desirable mechanical properties. Finally, our most recent work on GBE for the control of electrical resistivity in gold thin films is introduced as an example of a possible challenge toward GBE for high performance functional materials.

### Effect of grain boundary microstructure on hardness in electrodeposited nanocrystalline materials

#### Effect of grain boundary density on hardness

**Figure 1:**
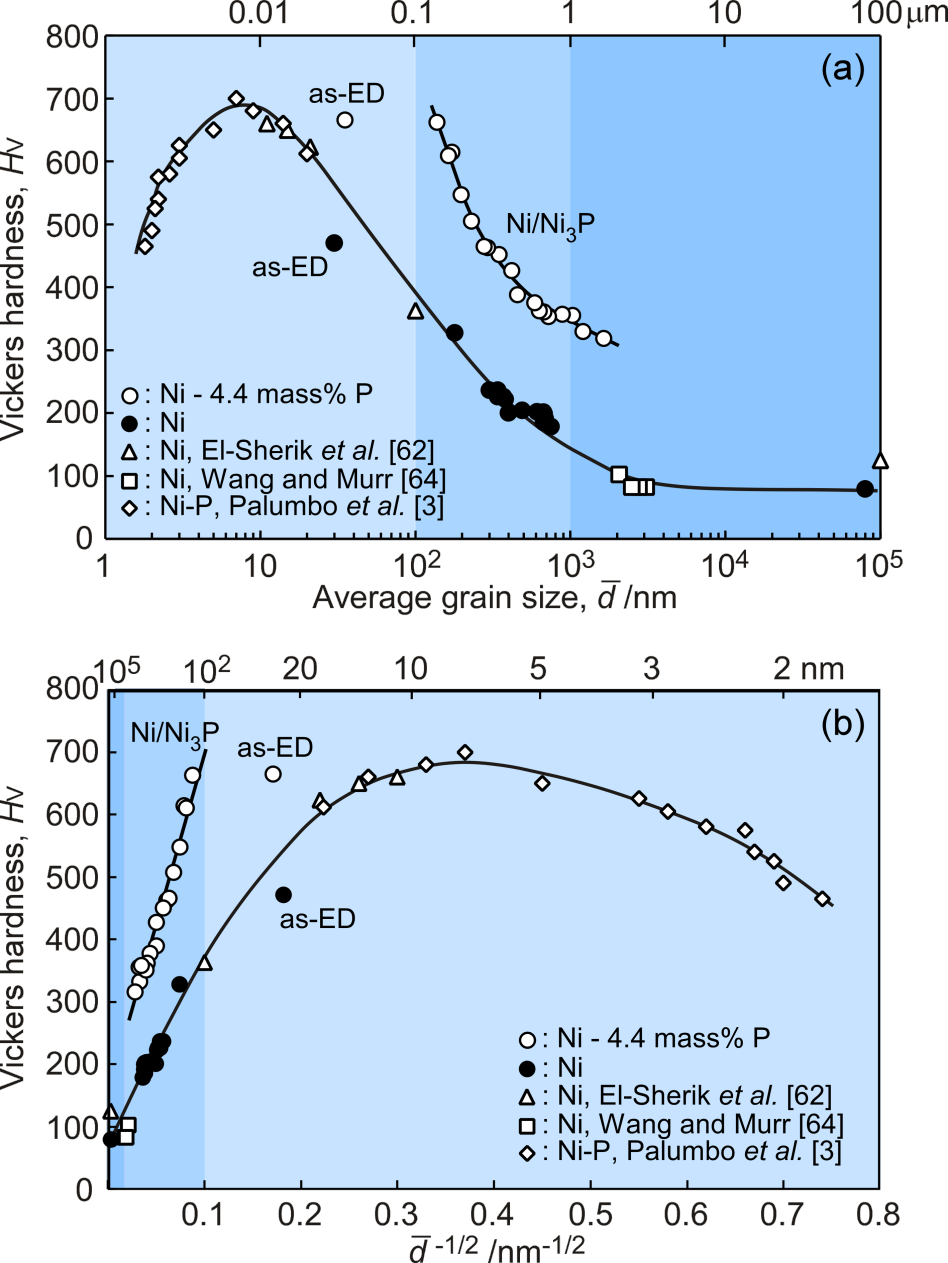
(a) The Vickers hardness as a function of the average grain size in electrodeposited and subsequently annealed Ni and Ni–P alloys. (b) The Hall–Petch plot of grain size dependence of the Vickers hardness.

As mentioned in the Introduction, nanocrystalline materials show considerably high strength and hardness, due to the presence of the extremely high density of grain boundaries and triple junctions, as well as other defects. Figure 1 shows the relationship between the Vickers hardness and the average grain size for pure nickel (Ni) and nickel–phosphorus (Ni–P) alloy specimens produced by electrodeposition and subsequent annealing. The data obtained from our recent investigation are shown together with those for pure Ni [62,64] and Ni–1.2 mass % P alloy [3] reported by other researchers. The state of the supersaturated solid solution in as-electrodeposited Ni–4.4 mass % P alloy specimens was confirmed, although the Ni–P phase diagram [65] indicates that the solubility limit of phosphorus into nickel matrix is 0.17 mass %. Accordingly, the Ni_3_P phase may precipitate in the Ni–4.4 mass % P alloy specimens during annealing. As shown in Figure 1a, the hardness drastically increased in the Ni and Ni–P alloy specimens with an average grain size of less than 10^3^ nm (1 μm), while the hardness gently increased in the specimen with conventional grain size. It has been reported that the density of grain boundaries and triple junctions also drastically increased in the materials with average grain size less than 1 μm [66]. Therefore, it is suggested that an increase of grain boundary density was essential for a considerably high strength and hardness in nanocrystalline and submicrometer-grained materials.

The Hall–Petch relationship between the hardness and the average grain size fails when the average grain size is much smaller than 30 nm, as shown in Figure 1b. It has been suggested that the dominant deformation mechanism in nanocrystalline materials with an extremely high density of grain boundaries is different in polycrystalline materials with conventional grain structure. Grain boundary sliding, grain boundary diffusion-controlled creep, and the contribution of triple line diffusion have been proposed as possible mechanisms of deformation in nanocrystalline materials [67].

It should be noted that the P composition strongly affects the hardness of nanocrystalline Ni. In as-electrodeposited Ni–4.4 mass % P alloy specimens, P atoms mostly segregate at grain boundaries, because of the low solubility limit (0.17 mass %) of P atoms in the Ni matrix and of a very high density of grain boundaries and triple junctions. Although the average grain size in as-electrodeposited pure Ni and Ni–4.4 mass % P alloy specimens is almost the same, the hardness of the as-electrodeposited Ni–4.4 mass % P alloy specimen increased by 40% compared to the as-electrodeposited pure Ni specimen. The grain boundary segregation of P atoms may result in the observed excess hardening of nanocrystalline Ni due to the enhancement of grain boundary hardening [68]. Moreover, the precipitation of the Ni_3_P phase affects the hardening of the Ni–P alloy. The positive Hall–Petch slope behavior between the hardness and average grain size was observed. The Ni/Ni_3_P alloy specimens prepared by annealing of electrodeposited Ni–4.4 mass % P solid solution show that the hardness of the Ni/Ni_3_P alloy more strongly depends on the grain size than in Ni specimens.

#### Structure-dependent grain boundary hardening and effects of GBCD and triple junction character distribution on the hardness

It has been revealed that the hardness locally increases around grain boundaries against the grain interior, depending on the grain boundary character [69–74] and the degree of grain boundary segregation [69,75–77]. The present authors have reported that the hardening ratio for neighboring grain interiors of specific types of grain boundaries can change and tend to be lower at low-angle boundary and low-Σ CSL boundaries than at random boundaries in polycrystalline molybdenum. The generated dislocations can transfer or pass across low-angle boundaries more easily than random boundaries composed of more complicated tilt and twist components at room temperature [72]. It is evident that the grain boundary character can strongly affect the hardness at individual grain boundaries in bicrystals and polycrystalline materials.

**Figure 2:**
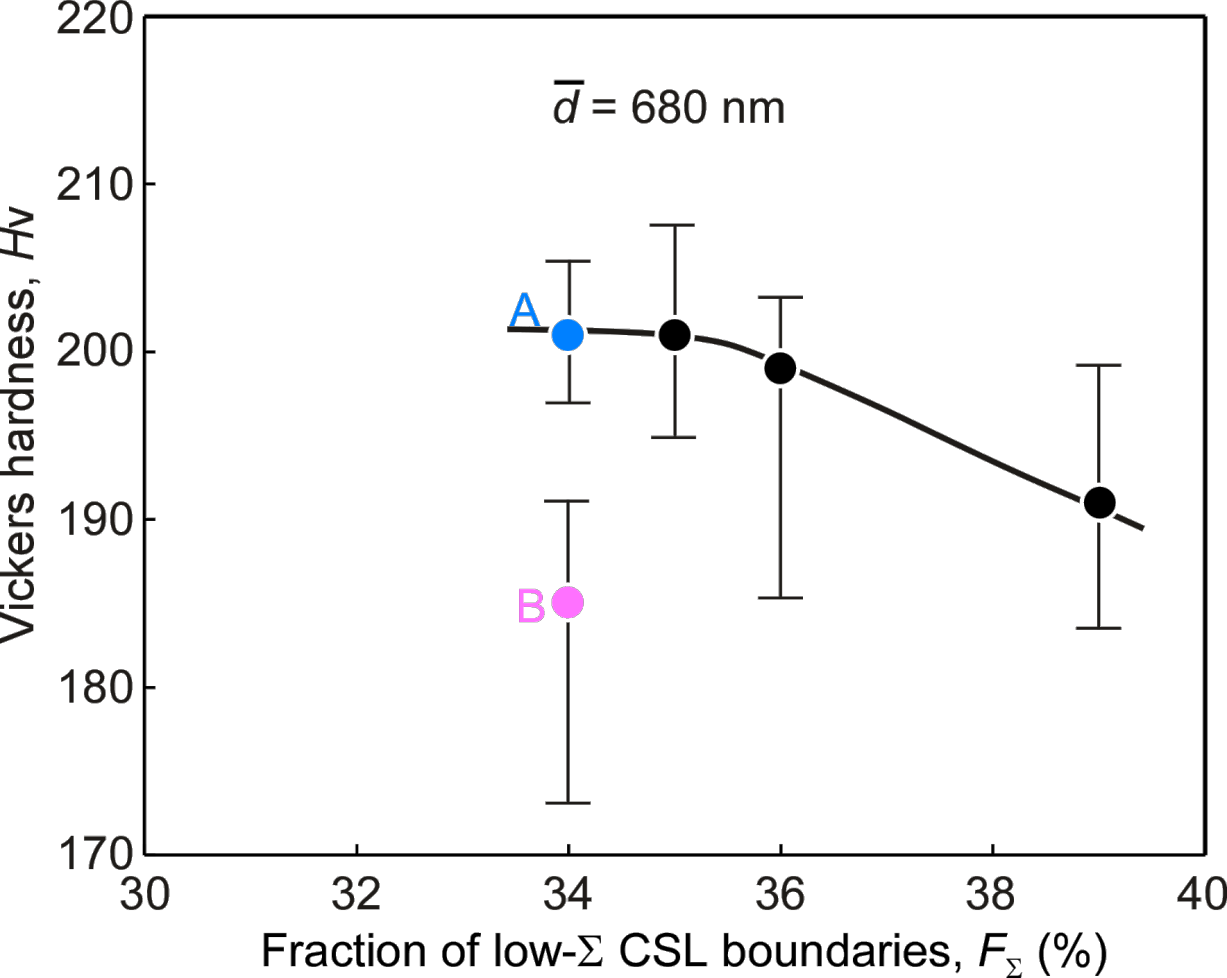
Relationship between the Vickers hardness and the fraction of low-Σ CSL boundaries for the submicrometer-grained Ni specimens with an average grain size of 680 nm.

Figure 2 shows the relationship between the Vickers hardness and the fraction of low-Σ CSL boundaries including low-angle (Σ1) boundaries in submicrometer-grained Ni specimens with the average grain size of 680 nm. These specimens were prepared by annealing at different temperatures from the electrodeposited nanocrystalline Ni specimens. The Vickers hardness decreased with increasing fraction of low-Σ CSL (Σ1–Σ29) boundaries for the studied specimens with almost the same average grain size. The hardness obviously decreased from 34 to 39%, with a slight change (5%) of the fraction of low-Σ CSL boundaries. Thus, it is evident that the GBCD-dependent hardness becomes more remarkable in nanocrystalline and submicrometer-grained materials in comparison with conventional polycrystalline materials.

Here, it should be noted that the specimens with the same fraction of low-Σ CSL boundaries of 34%, designated by A and B, showed quite different values of hardness, by almost 10%. The observed difference of the hardness may originate from other factors associated with grain boundary microstructure, that is, the triple point character. In order to clarify this, we further examined the grain boundary microstructure in the studied specimens. In principle, triple junctions are simply classified into four different types in terms of the connectivity of two different types of grain boundaries, i.e., random type and special low-Σ CSL boundaries, as discussed in [78] and [72,74]: (1) R0 type with no random boundaries, (2) R1 type with 1 random and 2 low-Σ CSL boundaries (including low-angle boundaries), (3) R2 type with 2 random and 1 low-Σ CSL boundaries, and (4) R3 type with 3 random boundaries.

**Figure 3:**
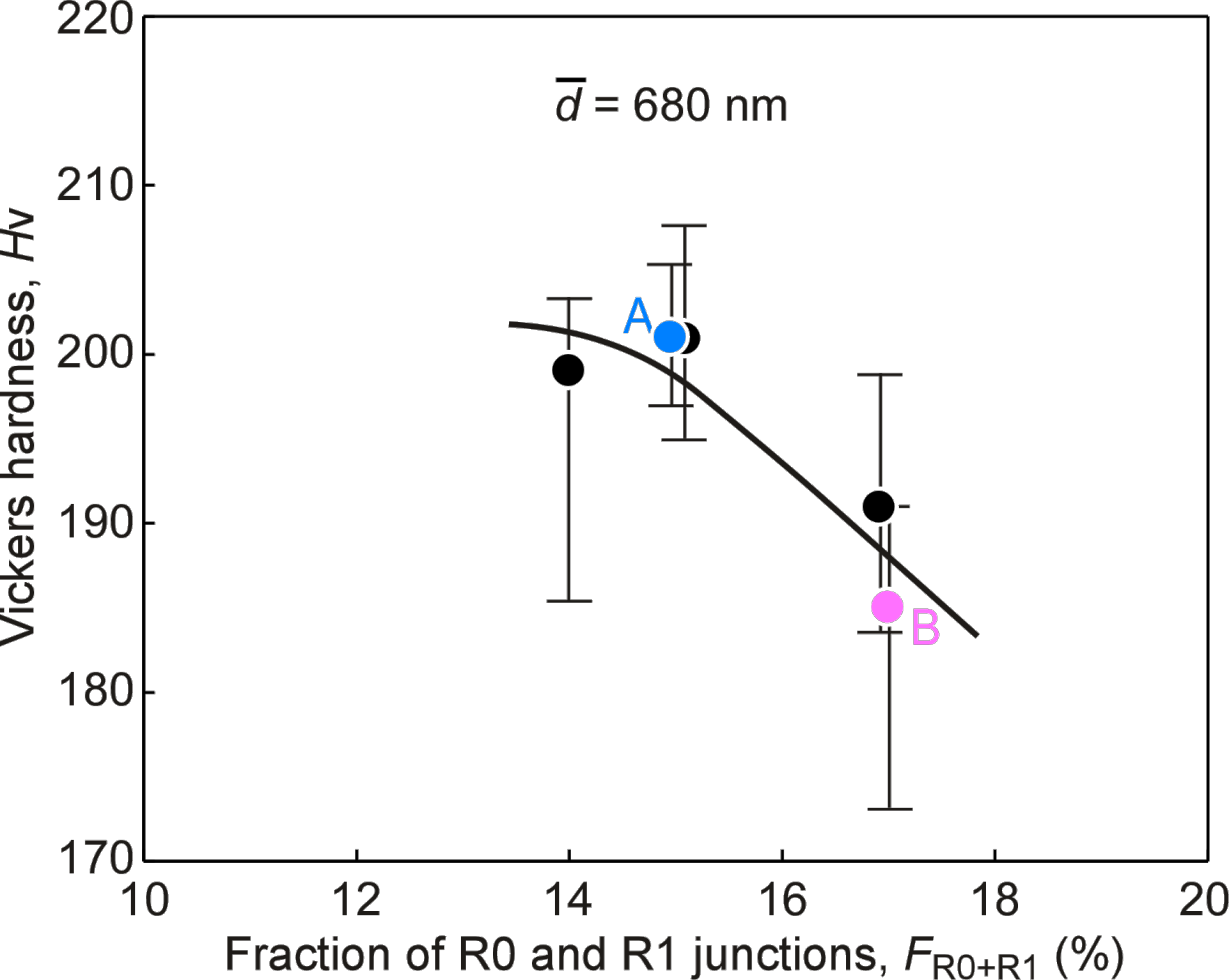
Relationship between the Vickers hardness and the fraction of R0 and R1 type triple junctions composed of less random boundaries in the submicrometer-grained Ni specimens.

Figure 3 shows the relationship between the Vickers hardness and the total fraction of specific types (R0 and R1) of the triple junctions observed in the same specimens corresponding to the specimens indicated in Figure 2. It was found that the hardness of the studied specimens clearly decreased with increasing total fraction of R0 and R1 type triple junctions with less random boundaries, and that the triple junctions with the higher connectivity of low-Σ CSL boundaries showed the lower triple junction hardening, as expected from the previous similar work for ordinary polycrystals [72]. Therefore, it is suggested that the triple junction character distribution (TJCD) also strongly affects the hardness of submicrometer-grained nickel specimens as well as GBCD. This is probably because the interaction of crystal dislocations with grain boundaries was found to strongly depend on the boundary character, leading to the passage of them across the boundaries, as discussed in detail in [79]. Accordingly, it is reasonable to understand that the TJCD plays an important role in the mechanical properties of nanocrystalline and submicrometer-grained materials with a very high density of triple junctions together with grain boundaries. However, a more precise understanding and control of grain boundary microstructures are necessary for further improvement of their mechanical properties and the generation of new functions in nanocrystalline materials with desirable bulk properties in future.

### GBE for control of segregation-induced embrittlement in nanocrystalline and submicrometer-grained Ni

Segregation-induced intergranular embrittlement is a serious problem that degrades the performance reliability of various types of structural materials. In nanocrystalline and submicrometer-grained materials possessing a very high density of grain boundaries and triple junctions, the detrimental effect of intergranular segregation seems to be more serious in comparison with conventional polycrystalline materials with the ordinary grain size ranging from the micrometer to the millimeter level. The amount of segregating impurity atoms at grain boundaries is well known to strongly depend on the grain boundary character and structure [69,80–84]. Bouchet and Priester [82–83] have found that the intergranular segregation of sulfur in Ni occurred preferentially at high-energy general random boundaries, but is very difficult or small at low-energy special boundaries. They suggested that the grain boundary plane orientation or low boundary planar atomic density can affect the amount of segregation even at such special CSL boundaries as Σ3, depending on either the coherent or incoherent part. As is clear from the general finding of structure-dependent grain boundary segregation, the segregation-induced intergranular embrittlement should become more serious in nanocrystalline materials and needs to be effectively controlled through GBE.

**Figure 4:**
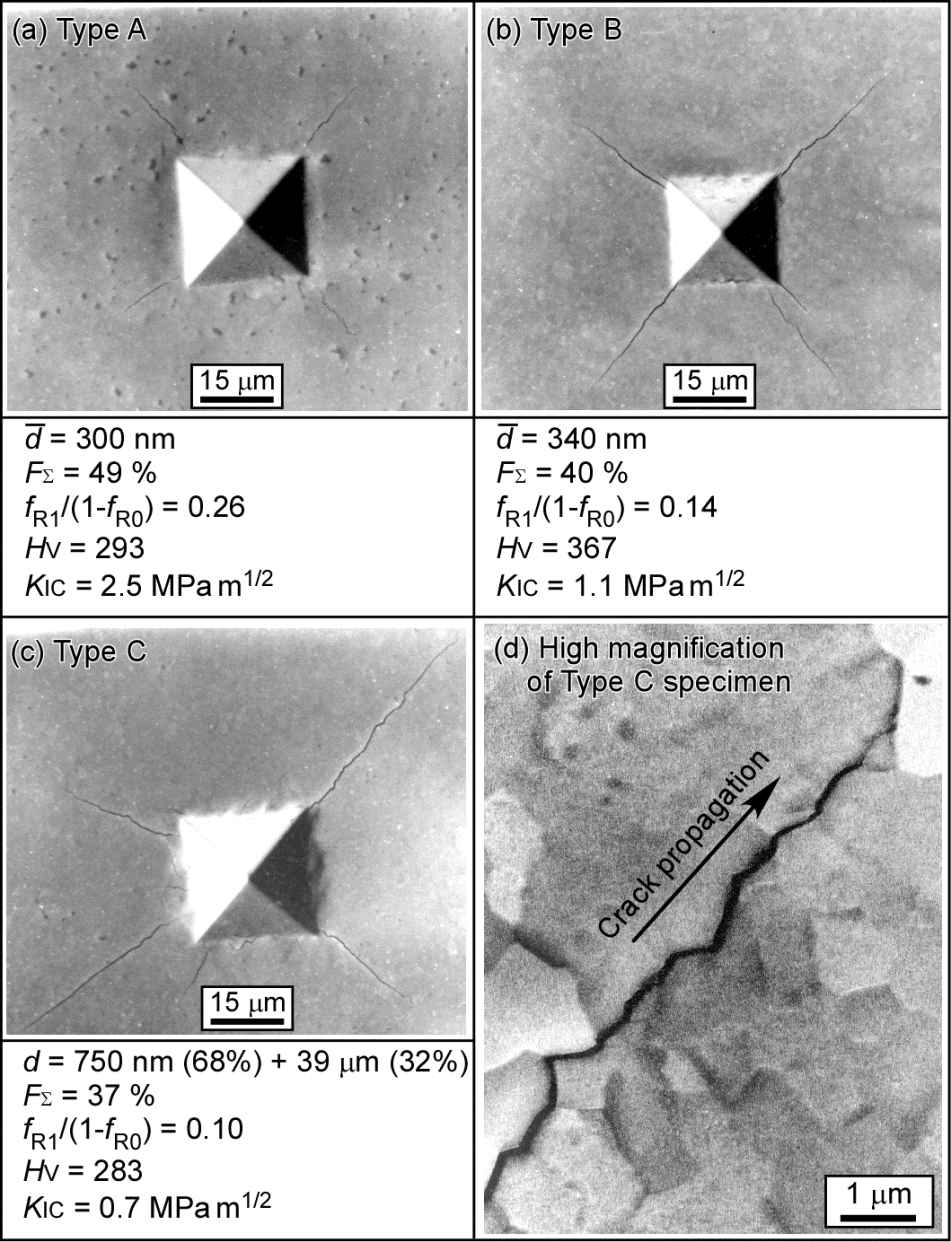
SEM micrographs of cracks introduced by indentation tests in the sulfur-doped fine-grained Ni specimens with different grain boundary microstructures. Type A and Type B specimens have similar average grain size, but different fractions of low-Σ CSL boundaries of 49 and 40%, respectively (a,b). Type B and Type C specimens have similar GBCD, but different grain sizes of 340 nm and 750 nm and 39 μm, respectively (b,c). The SEM micrograph of the crack path in the Type C specimen exhibiting coarse grains (d) [85]. Figure reprinted with permission from [85], copyright 2010 Elsevier Ltd.

Now let us look at the results on the GBCD-dependent fracture process in submicrometer-grained materials. Figure 4 shows SEM micrographs of the propagation path of cracks produced by Vickers indentation tests at a load of 1.96 N for the sulfur-doped submicrometer-grained Ni specimens with different grain boundary microstructures [85]. Type A and Type B specimens had different fractions of low-Σ CSL boundaries (including low-angle boundaries) of 49 and 40%, but almost the same average grain size of 300 and 340 nm, respectively. It was found that the crack length from the tip of indentation in the Type A specimen with a higher fraction of low-Σ CSL boundaries (*F*_Σ_ = 49%) was shorter than in the Type B specimen with a lower fraction of low-Σ CSL boundaries (*F*_Σ_ = 40%). The fracture toughness *K*_IC_ measured by indentation fracture (IF) method for the Type A and the Type B specimens were 2.5 MPa m^1/2^ and 1.1 MPa m^1/2^, respectively. Evidently, the fracture toughness of the Type A specimen with a higher fraction of low-Σ CSL boundaries is more than twice higher when the fraction of low-Σ CSL boundaries was increased by about 10%. This was because the crack that preferentially propagated along weak random boundaries with preferential S-segregation in the Ni–S alloy specimen, as shown in Figure 4d. The fraction and the connectivity of fracture-resistant low-Σ CSL boundaries, or crack-leading weak random boundaries are key parameters in controlling the typical percolation-dependent intergranular brittle fracture mode, or combined mode of mixture of intergranular and transgranular fracture. This finally leads to the characteristic bulk fracture properties in submicrometer-grained Ni. The more precise control of the occurrence of segregation-susceptive grain boundaries (high-energy random boundaries) by GBE based on the percolation-dependent fracture must be necessary for the control of segregation-induced intergranular embrittlement.

Along this line, the fraction of percolation-resistant triple junctions, *f*_R1_/(1−*f*_R0_), was evaluated, following Kumar et al. [86], as shown in Figure 4. The values of *f*_R1_/(1−*f*_R0_) were 0.26 and 0.14 for the Type A and the Type B specimens, respectively. Accordingly, the crack propagation in the Type A specimen was probably inhibited by the combined effects originating from the high fractions of low-Σ CSL boundaries and percolation-resistant triple junctions. As a result, the fracture toughness drastically increased for the Type A specimen. The Vickers hardness of the Type A specimen (*H*_V_ = 293) is lower than that of the Type B specimen (*H*_V_ = 367), because the degree of grain boundary hardening at low-Σ CSL boundaries is lower than that at random boundaries, as discussed in the previous section. This suggests that there exists some plasticity during the dislocation-boundary interaction.

It is worth noting that the crack length in the Type C specimen with duplex grain structure with different grain sizes of 750 nm and 39 μm was longer than that in the Type B specimen, despite having almost the same fraction of low-Σ CSL boundaries in the Type C and Type B specimens. The fracture toughness *K*_IC_ was 1.1 MPa m^1/2^ and 0.7 MPa m^1/2^, for the Type B and the Type C specimens, respectively. The fraction of percolation-resistant triple junctions, *f*_R1_/(1−*f*_R0_) was 0.14 and 0.10, in the Type B and the Type C specimens, respectively. Therefore, the crack propagation can be prevented more effectively in the Type B specimen. On the other hand, a crack nucleated at a random boundary of coarse grain can readily propagate further at longer, random boundaries until it reaches the percolation-resistant triple junction in the Type C specimen composed of a mixture of coarse and fine grains. These findings confirm that segregation-induced embrittlement in polycrystalline materials can be well controlled by optimizing the grain boundary microstructure, especially by GBCD, the grain boundary connectivity, and the heterogeneity of the grain size distribution. This is true even when local in nature, as revealed by our recent work on sulfur-doped polycrystalline Ni [87].

### Effects of grain boundary microstructure on fatigue deformation and fracture in nanocrystalline Ni–P alloy

In recent years, it has been revealed that grain boundaries play important and different roles in fatigue crack nucleation [88–97] and propagation [44–45,98–99] in polycrystalline materials, depending on the grain boundary character and structure. The present authors have confirmed that intergranular fatigue cracks preferentially nucleate along random boundaries in polycrystalline aluminum, while they do not nucleate along low-angle boundaries [97]. The low-Σ CSL boundaries show the higher resistance to fatigue cracking than the random boundaries [97], although the preferential nucleation at coherent twin boundaries, namely {111}/Σ3 CSL boundaries, were previously reported for face-centered cubic (FCC) materials such as copper [90–91].

In the case of nanocrystalline and submicrometer-grained materials, it has been suggested that the roles of grain boundaries in fatigue deformation and fracture become more important due to the instability of grain boundary microstructure [100–106]. However, unfortunately, the effect of the instability of grain boundary microstructure on fatigue deformation and fracture in nanocrystalline materials has not been fully studied and understood yet, despite extensive previous works on the fatigue property in nanocrystalline materials [107–110]. It is difficult to fully understand the characteristics and dominant mechanisms of fatigue deformation and fracture in nanocrystalline materials without fundamental knowledge of microstructural change, especially grain boundary microstructure during cyclic deformation, resulting in the instability of grain boundary microstructure characterized by several key parameters, mentioned already.

**Figure 5:**
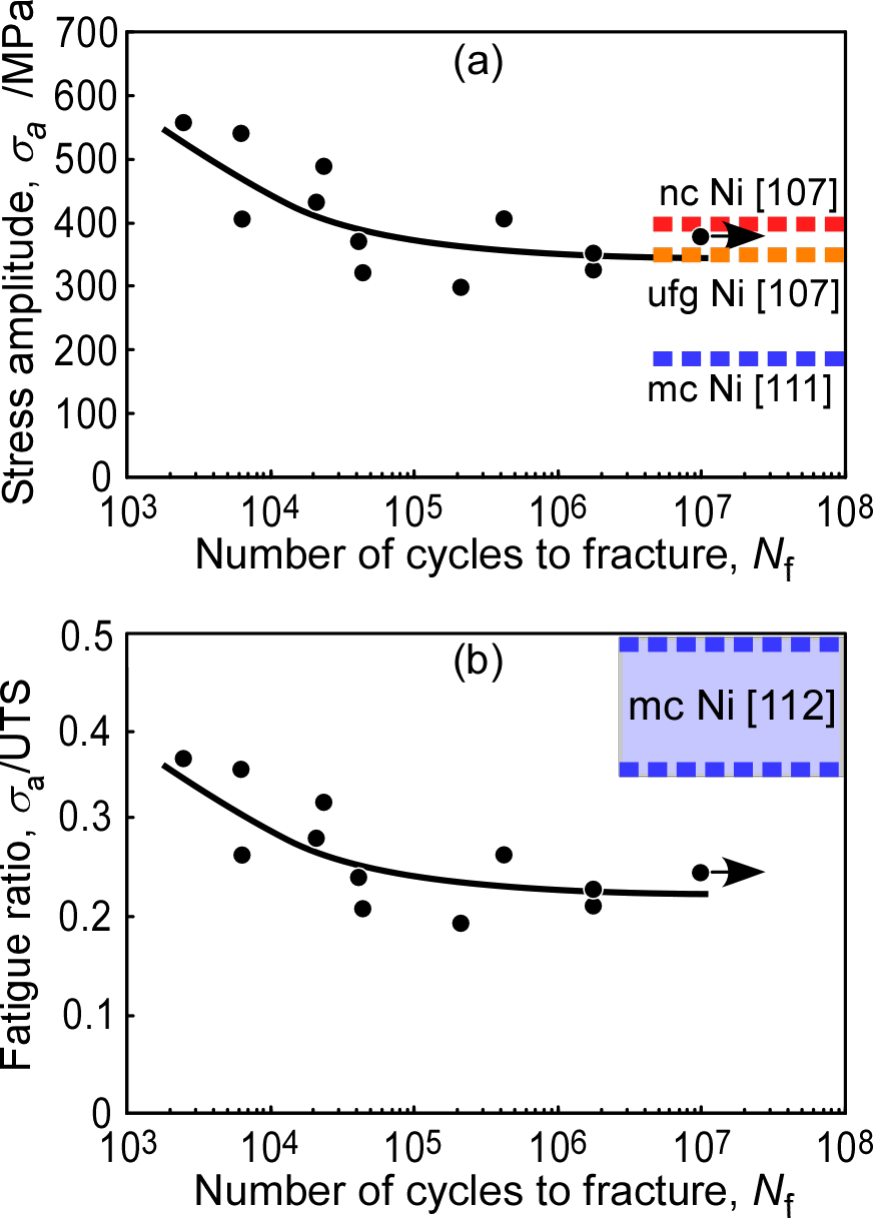
*S*–*N* curves of nanocrystalline Ni–2.0 mass % P alloy specimens: (a) stress amplitude versus logarithm of number of cycles to fracture [110] and (b) stress amplitude normalized by ultimate tensile strength (fatigue ratio) versus logarithm of number of cycles to fracture. Figure reprinted with permission from [110], copyright 2009 Elsevier Ltd.

Figure 5a shows the *S*–*N* curve which indicates the relationship between the stress amplitude and number of cycles to fracture in electrodeposited nanocrystalline Ni–2.0 mass % P alloy specimens with the initial average grain size of 45 nm [110]. The fatigue limit data are shown in Figure 5a together with those taken from the literature for electrodeposited nanocrystalline Ni with the average grain size of 20 nm [107], for ultrafine-grained nickel with the average grain size of 300 nm [107] and for electrodeposited microcrystalline nickel with conventional grain size [111]. The fatigue limit of about 360 MPa estimated for the Ni–P alloy specimens was two times higher than that of the microcrystalline nickel with conventional grain size. This estimated value of fatigue limit was close to the data reported for ultrafine-grained Ni specimens, and lower than for nanocrystalline Ni with the average grain size of 20 nm.

Figure 5b shows the *S*–*N* curve indicating the relationship between the stress amplitude normalized by the ultimate tensile strength (fatigue ratio, σ_a_/UTS) and number of cycles to fracture (*N_f_*). It was found that the fatigue limit for Ni–2.0 mass % P alloy specimens was about 23% of their ultimate tensile strength of 1550 MPa. The fatigue ratio of the fatigue limit of conventional polycrystalline Ni ranged between 0.35 and 0.50 [112]. Thus, it was found that the enhanced ratio of the fatigue limit was limited to a lower degree in nanocrystalline Ni–2.0 mass % P alloy. This finding is very interesting and deserves to be explored in a more detailed investigation.

**Figure 6:**
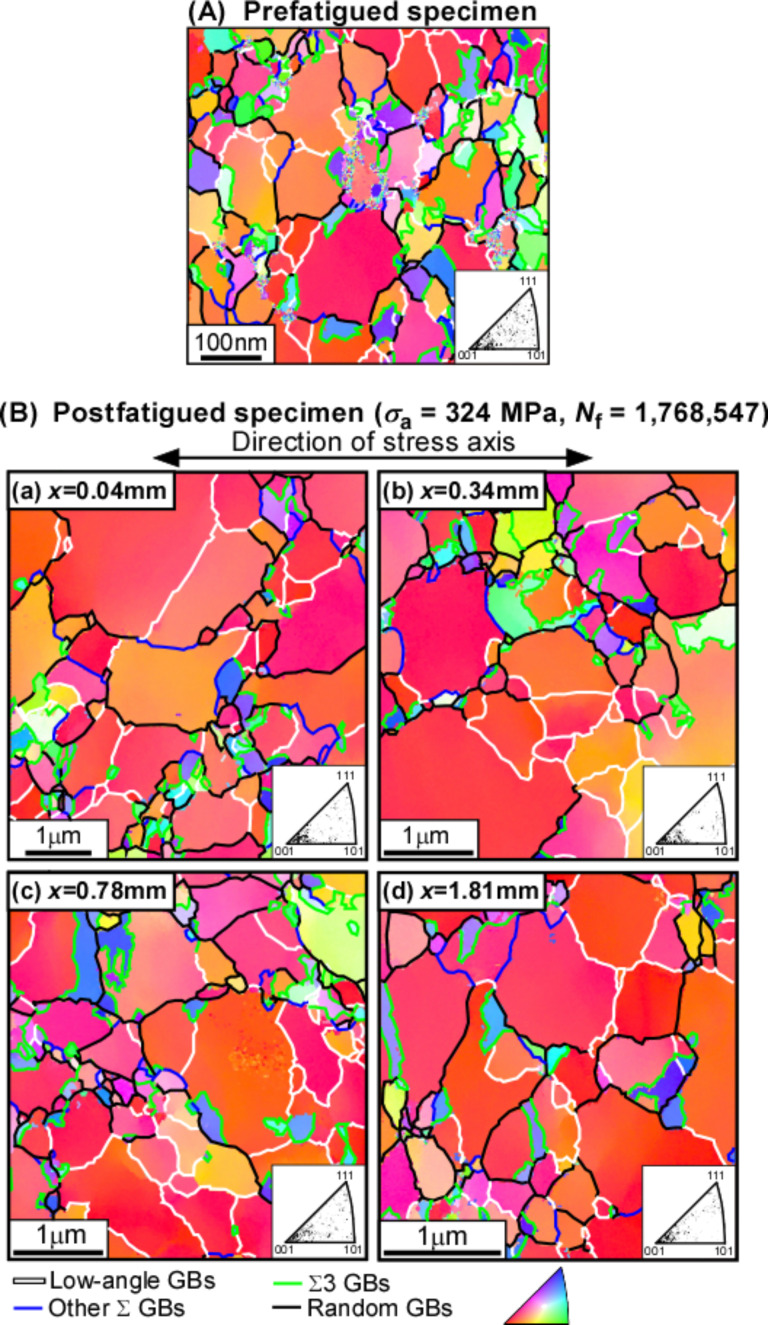
OIM micrographs with inverse pole figures (IPF) of grain orientation distribution for (A) prefatigued and (B) postfatigued Ni–2.0 mass % P alloy specimens [113]. Published by Elsevier Ltd. Figure reprinted with permission from [113], copyright 2015 Elsevier Ltd.

Quite recently, the present authors have found from more detailed SEM/electron backscattered diffraction (EBSD)/orientation imaging microscopy (OIM) observations during fatigue deformation that the low fatigue ratio for the fatigue limit in nanocrystalline Ni–2.0 mass % P alloy resulted from the instability of the grain boundary microstructure [113]. Figure 6a,b shows the OIM micrographs with the inverse pole figures inserted at the bottom corner for the prefatigued specimen and for the postfatigued specimens of Ni–2.0 mass % P alloy [113]. The grain and grain boundary microstructure drastically changed from those of initially nanocrystalline grain structure in the prefatigued specimen (Figure 6a) to the fine-grained structure with submicrometer average grain size during high cycle fatigue test, although the sharpness of the {001} texture hardly changed among the pre- and postfatigued specimens. It should be noted that the trace of grain boundaries exhibits an interesting characteristic feature that grain boundaries are migrated and aligned at about 45° against the stress axis. This results in the evolution of a “diamond-shaped” grain structure, which was observed in conventional polycrystalline materials during cyclic deformation at high temperatures [114–117]. The grain growth by high-cycle fatigue may result from a rapid migration of low-angle boundaries involving some dislocation mechanisms and enhanced by segregated P atoms at finally resultant random boundaries along shear bands. The operating mechanism will be explained later in some detail.

**Figure 7:**
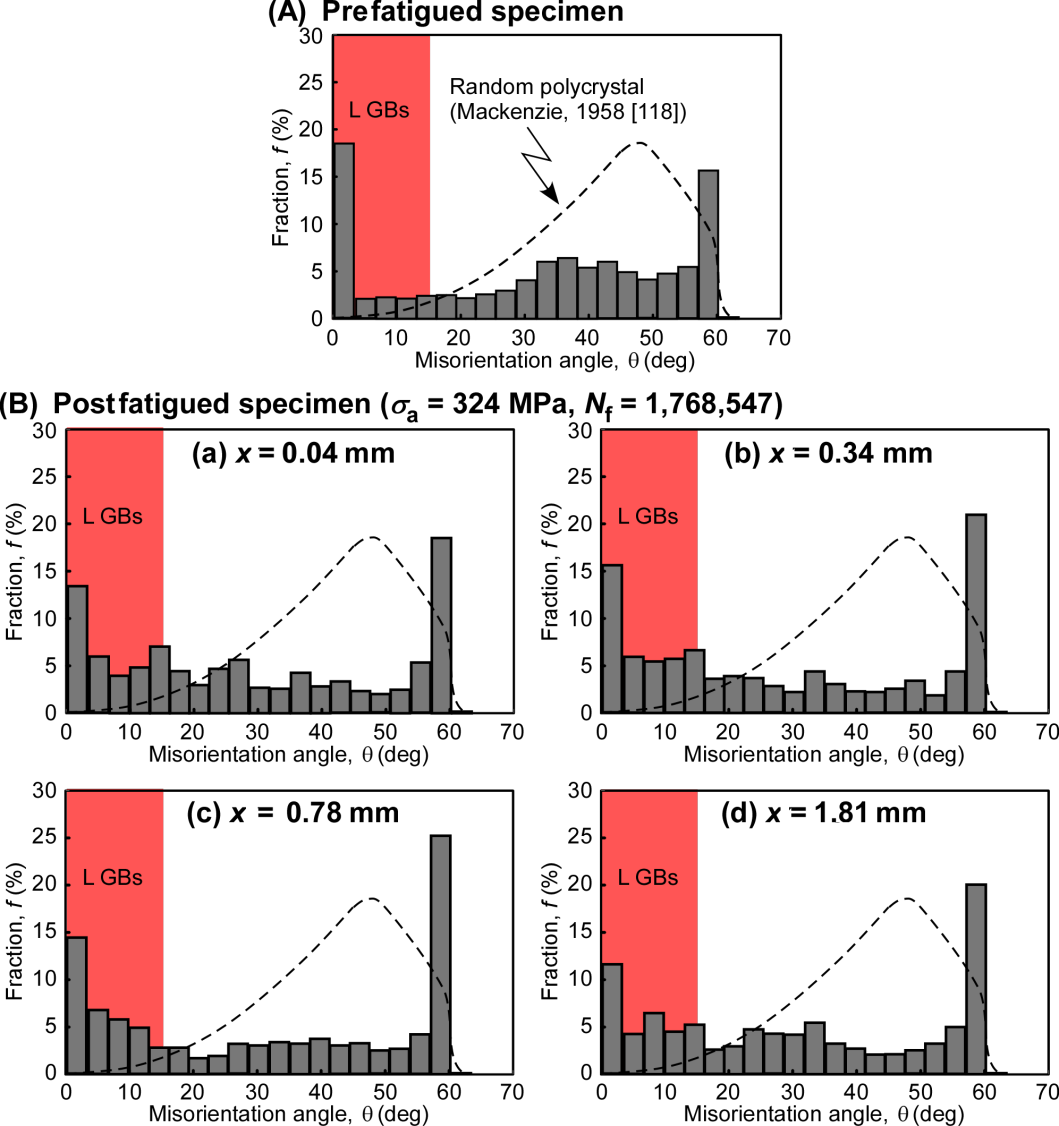
Misorientation angle distributions for (a) prefatigued and (b) postfatigued Ni–2.0 mass % P alloy specimens, where *x* indicates the position from the fracture surface [113]. Figure reprinted with permission from [113], copyright 2015 Elsevier Ltd.

**Figure 8:**
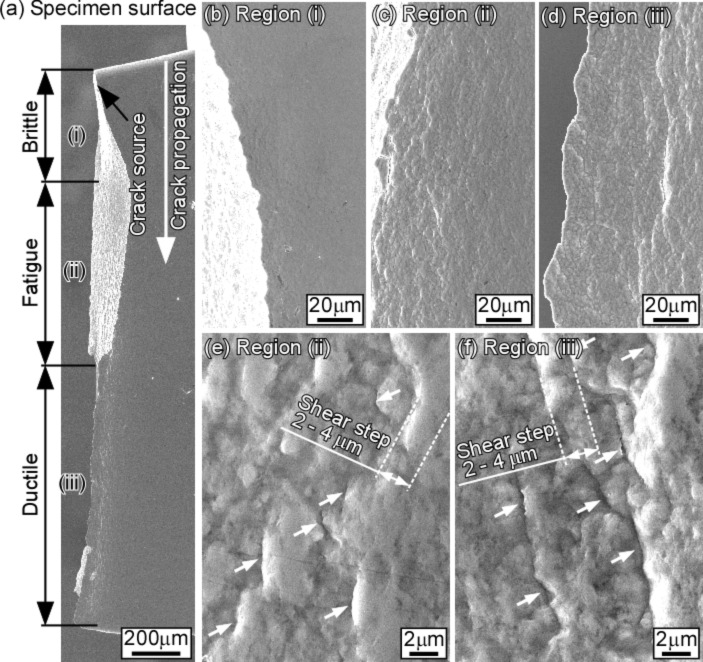
Specimen surface of electrodeposited nanocrystalline Ni–2.0 mass % P alloy specimen after high-cycle fatigue test: (a) low-magnification image of the whole fracture surface; (b–d) are medium-magnification images and (e–f) are high-magnification images of areas corresponding to the regions (i), (ii) and (iii), respectively [113]. Figure reprinted with permission from [113], copyright 2015 Elsevier Ltd.

Figure 7 shows the misorientation angle distributions of grain boundaries before and after fatigue deformation involving the cyclic stress-induced grain growth [113]. The misorientation angle distribution for a random polycrystal, theoretically predicted by Mackenzie, is shown by the dotted curve in this figure [118]. A certain fraction of low-angle boundaries with a misorientation angle lower than 3° in the prefatigued specimen (Figure 7a) transformed into the grain boundaries having the misorientation angles 3° < θ < 25° in the postfatigued specimen (Figure 7b). A higher fraction of Σ3 CSL boundaries hardly changed during fatigue. The cyclic stress-induced grain growth, accompanying the transformation of low-angle boundaries into the boundaries with higher misorientation angle, is associated with the evolution of a “diamond-shaped” grain structure along initially formed shear bands. This resulted in intergranular fatigue fracture due to grain boundary sliding, as can be seen from the fracture surface of fatigue fractured nanocrystalline Ni–2.0 mass % P alloy specimens (Figure 8). It is surprising to see that, as indicated in Figure 7b, there was no large difference of the misorientation angle distribution as a function of the distance from the position of the main crack in the postfatigued specimen. This suggests that more homogeneous fatigue deformation is assisted by dynamic grain growth.

**Figure 9:**
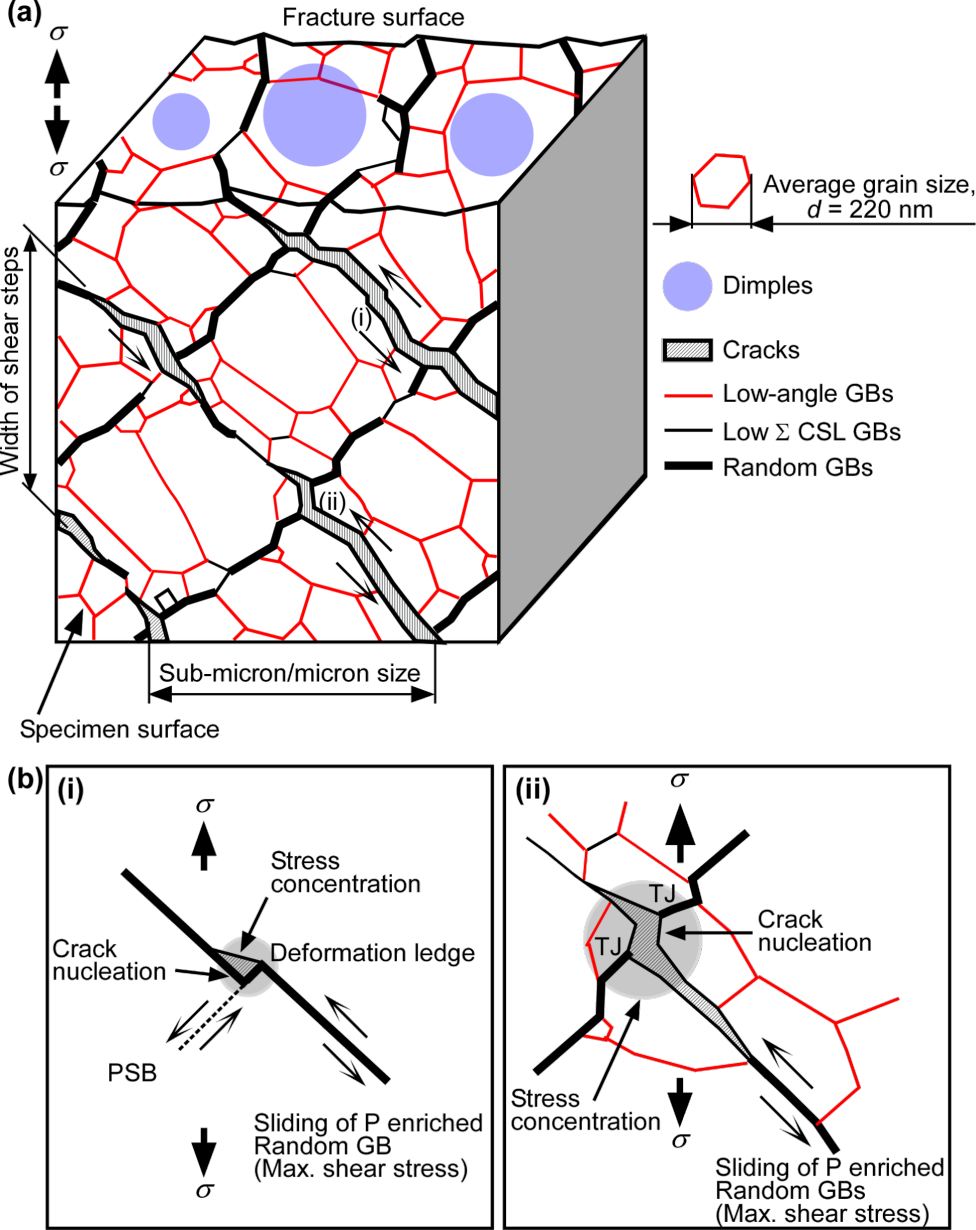
(a) Schematic illustration of the mechanism of intergranular fatigue fracture at random boundaries and the formation of morphological features of the specimen surface and fracture surface associated with propagation of intergranular fatigue cracks in the nanocrystalline Ni–2.0 mass % P alloy specimen during high-cycle fatigue. (b) Possible mechanism of fatigue crack nucleation at random boundaries by (i) the formation of deformation ledge and (ii) the stress concentration of the triple junctions composed of random boundaries [113]. Figure reprinted with permission from [113], copyright 2015 Elsevier Ltd.

Figure 9 shows the schematic illustrations of the possible mechanism of intergranular fatigue fracture assisted by the cyclic stress-induced grain growth and the grain boundary configuration forming the “diamond-shaped” grain structure. The details of the proposed mechanism of grain growth-assisted fatigue intergranular fracture can be obtained from the original article [113].

The formation of a large width of striations and large size of dimples was often observed in the fracture surface of fatigued nanocrystalline metals and alloys [102,110,113,119] in relation to the presence of the {001} grain clusters. The {001} grain clusters interconnected by low-angle boundaries (indicated by white lines in Figure 6b) were probably deformed by shear stress as in the case of a single crystal, because the persistent slip bands (PSBs) can continuously transfer across the low-angle boundaries [97].

The fatigue cracks preferentially nucleated along random boundaries whose boundary plane may almost correspond to the direction of shear band. They nucleate at the deformation ledge produced at sliding random boundaries by the interaction with PSBs or triple junctions of high connectivity of random boundaries, as discussed in detail by Watanabe [120]. In fatigue fracture of nanocrystalline Ni, Kumar et al. [121] also reported the formation mechanism of deformation ledge, although the stress-induced grain growth and arrangement of random boundaries toward 45° to the stress axis was not observed in their nanocrystalline Ni specimens. Our recent observations strongly suggested the important roles of gran boundary microstructure in fatigue property and fracture behavior in nanocrystalline materials.

### A new approach to GBE based on fractal analysis of grain boundary microstructures in nanocrystalline materials

This section concerns the bulk mechanical properties of nanocrystalline materials, especially focusing on the intrinsic and extrinsic brittleness of grain boundaries in different environments. For this purpose, a new approach to GBE based on the fractal analysis of grain boundary microstructures is needed. This must take into account the structure-dependent intrinsic grain boundary properties in order to produce polycrystalline and nanocrystalline materials with high performance and desirable bulk properties. The effectiveness of individual grain boundaries is required to be quantitatively evaluated based on the fundamental data obtained from systematic works by using orientation-controlled bicrystals of metallic, intermetallic, semiconductor and ceramic materials. In order to evaluate the effects of the grain boundary microstructures on bulk properties of polycrystals, we need to reveal the characteristic features of grain boundary microstructures produced by applying various kinds of materials processing in 2D (thin films) or 3D bulky conventional polycrystalline materials.

Here we take two examples of a new approach to GBE based on fractal analysis of grain boundary microstructures in nanocrystalline materials. One is GBE for control of segregation-induced intergranular brittle fracture, the other is GBE for improvement of corrosion resistance in existing stainless steels, as a typical engineering metallic material with enhanced corrosion resistance. This kind of GBE has been originally attempted by us quite recently and provided us important clues to future development of GBE. First, let us explain the basic method of fractal analysis of grain boundary microstructures in polycrystalline materials. Here, we briefly mention the basis of the fractal analysis of grain boundary microstructure in a real polycrystalline specimen [87,122].

**Figure 10:**
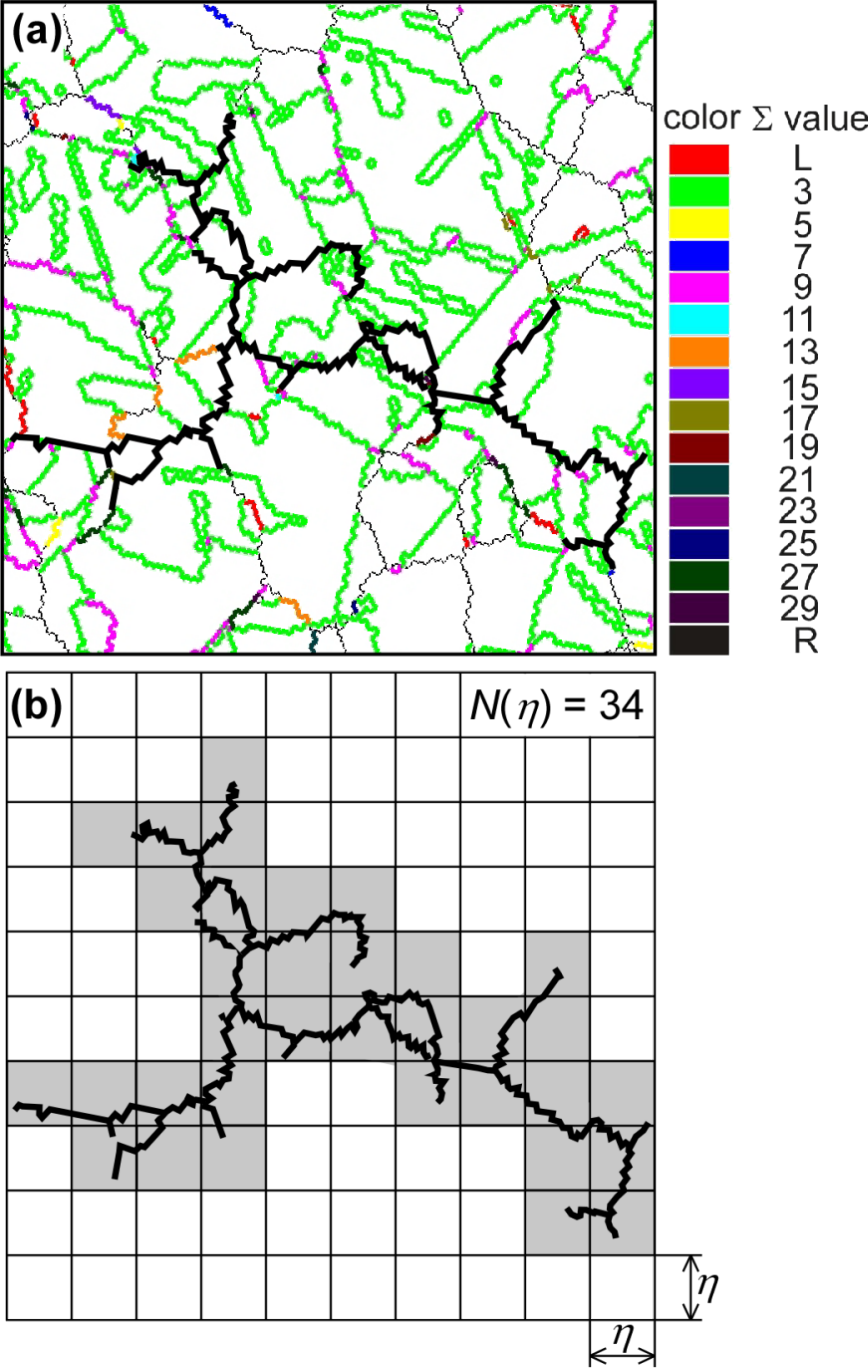
(a) Definition of the fractal dimension of the maximum random boundary connectivity (MRBC) and (b) demonstration of fractal analysis of MRBC by the box counting method [122]. Figure reprinted with permission from [122], copyright 2016 Elsevier Ltd.

In our investigations, the fractal analysis was carried out by the box-counting method for the random boundary network containing the maximum connectivity of random boundaries (maximum random boundary connectivity, MRBC) in the grain boundary map obtained from SEM/EBSD analysis on the specimen surface [87]. Figure 10a,b shows an example of the set of grain boundary map and the corresponding fractal trace of MRBC determined for a SUS316L austenitic stainless steel specimens [122]. The whole observed area is covered by the square box net with a given unit size, η.

**Figure 11:**
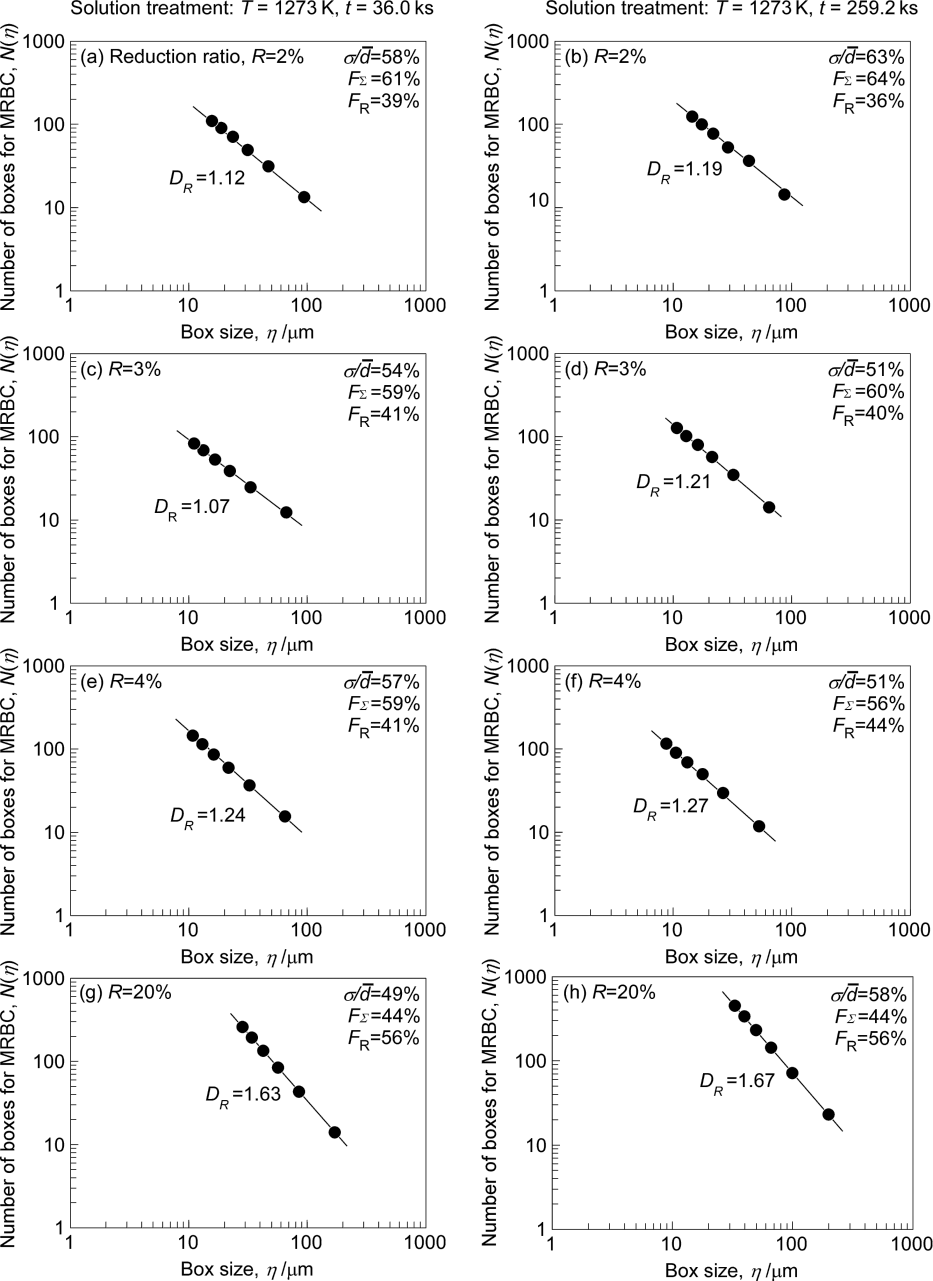
Relationship between the number of boxes *N*(η) for complete coverage of the maximum random boundary connectivity and the box size η for the SUS316L specimens subjected to different thermomechanical processing conditions of (a–h) [122]. Figure reprinted with permission from [122], copyright 2016 Elsevier Ltd.

**Figure 12:**
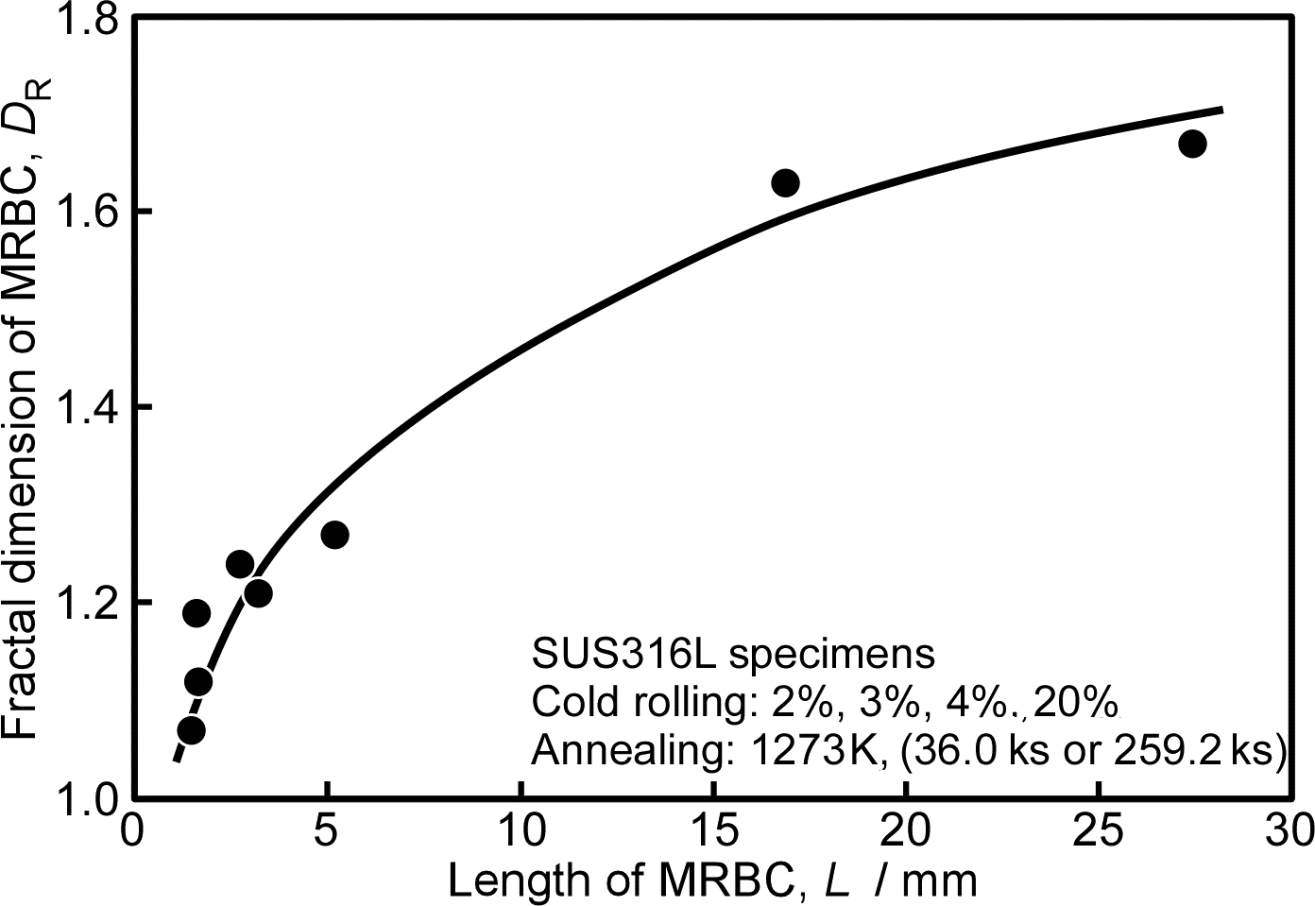
Relationship between the fractal dimension of MRBC and the length of MRBC.

Figure 11 shows the double-logarithm plots of the number of boxes *N*(η) for complete coverage of the MRBC and the box size η for the SUS316L specimens produced by thermomechanical processing in the different process conditions [122]. The value of *N*(η) was found to be a linear function of η. These results from different specimens indicate that the MRBC for different grain boundary microstructures in SUS316L steel specimens is of fractal nature. The fractal dimensions *D*_R_ evaluated by the slope of the log *N*(η) versus log η were found to change systematically from 1.07 to 1.67 for the studied SUS316L steel specimens. Namely, the fractal dimensions show a reasonable correlation with the total length of random boundary network, as seen from Figure 12. The longer percolation path composed of intergranular corrosion susceptive, random boundaries is characterized by the higher fractal dimension of MRBC. This finding has provided us with experimental evidence that the fractal dimension for MRBC, *D*_R_ is useful as a tool for quantitative evaluation of the random boundary connectivity controlling the intergranular corrosion susceptibility in polycrystalline materials.

**Figure 13:**
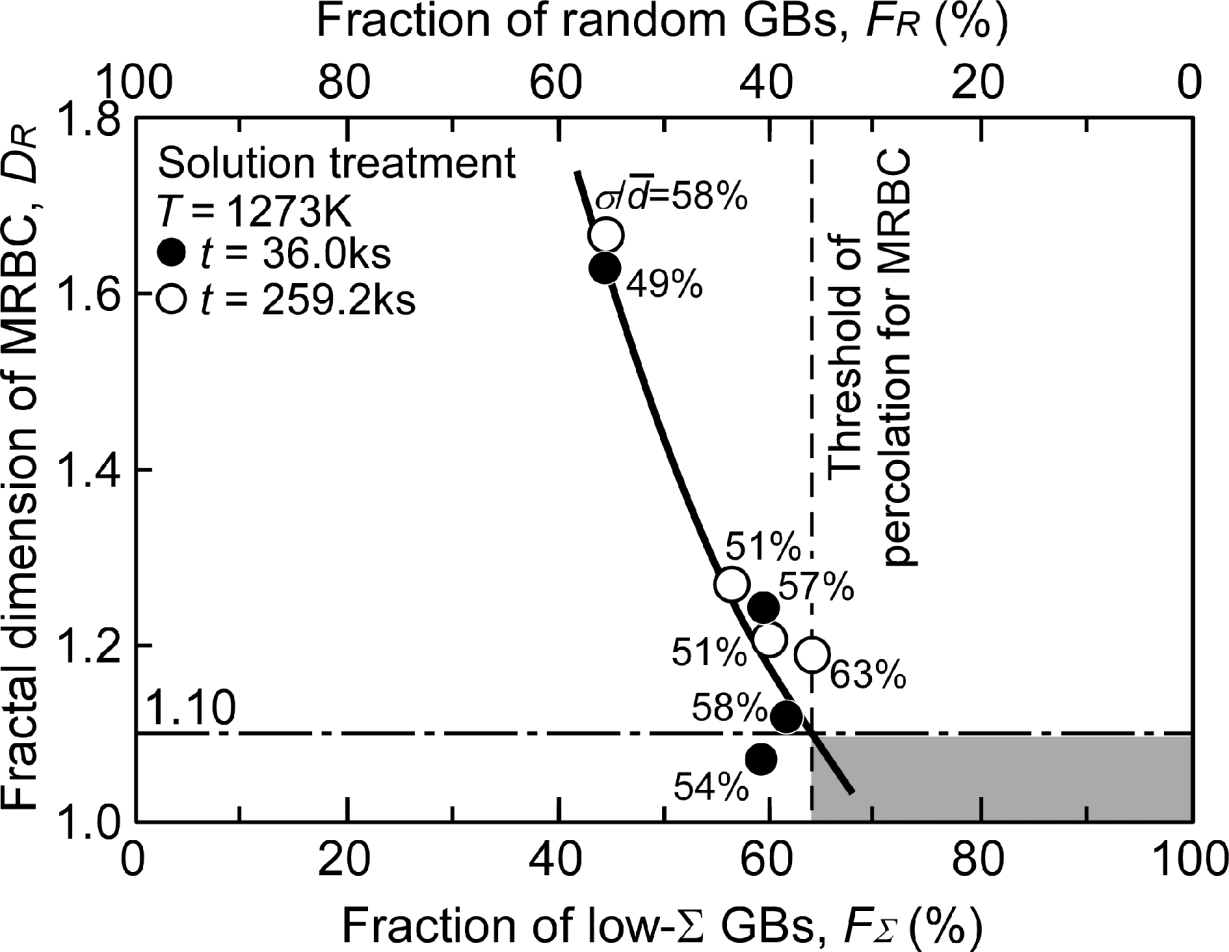
Relationship between the fractal dimension of the MRBC and the fraction of low-Σ boundaries *F*_Σ_ or random boundaries *F*_R_ for SUS316L specimens [122]. Figure reprinted with permission from [122], copyright 2016 Elsevier Ltd.

Figure 13 shows the relationship between the fractal dimension for MRBC, *D*_R_ and the fraction of random boundaries, *F*_R,_ or low-Σ CSL boundaries, *F*_Σ_ [122]. The coefficients of variation of the grain size distributions, 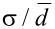 are indicated together with the data points in the figure. The value of *D*_R_ tends to decrease monotonically with decreasing value of *F*_R_ or with increasing value of *F*_Σ_. The larger value of 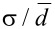 tends to generate the larger value of *D*_R_, even if the value of *F*_R_ or *F*_Σ_ would be kept similar, suggesting the path of percolation is more irregularly bent depending on the connectivity of random weak boundaries. The fractal dimension for MRBC, *D*_R_ may include the effect of a spread of the grain size distribution together with the effect of GBCD.

From the result shown in Figure 13, the value of *D*_R_ was estimated at about 1.10, by extrapolating the fitting curve to the experimental data up to the threshold value of *F*_Σ_ (65%) for the percolation-resistant low-Σ boundaries, or *F*_R_ (35%) for the percolation-assisting random boundaries. In the case of the SUS316L specimen hiving low *F_R_* and low *D*_R_, the curve runs into the shaded area in Figure 13. This suggests the intrinsically high corrosion resistance associated with GB microstructure. This is because of an interruption of the corrosion path along the random boundary network by the arrangement of corrosion-resistant low-Σ boundaries.

**Figure 14:**
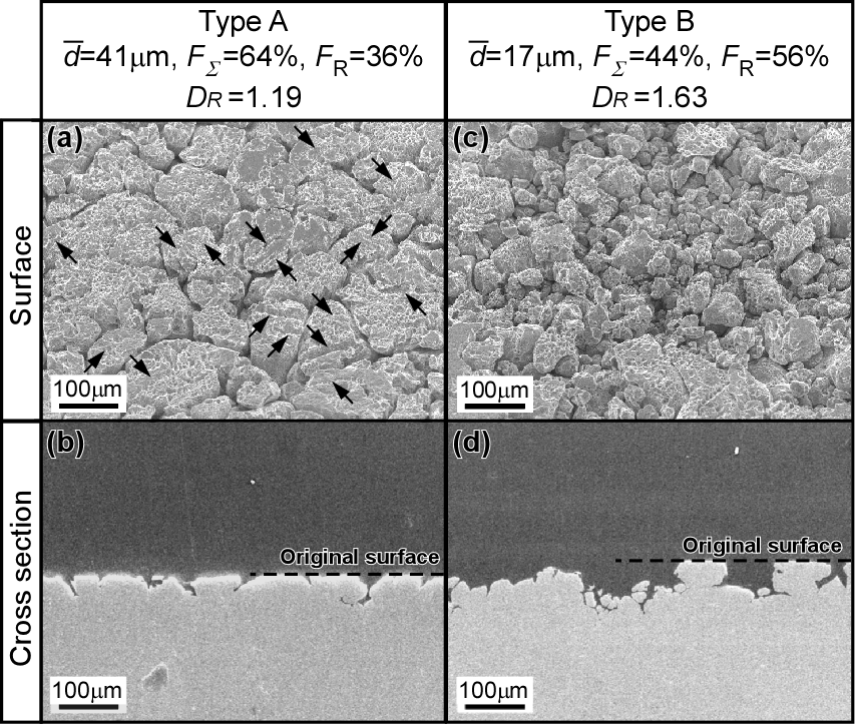
SEM micrographs of the surface (a,c) and the cross section (b,d) for the corroded specimens with different levels of the fractal dimension of MRBC. The black arrows show the position of annealing twin boundaries, namely {111}/Σ3 boundaries [122]. Figure reprinted with permission from [122], copyright 2016 Elsevier Ltd.

Figure 14 shows the SEM micrographs taken from the specimen surface and the vertical cross section for the two corroded SUS316L specimens with ordinary grain sizes and different values of the fractal dimension of MRBC, *D*_R_ [122]. Type A specimen had an average grain size of 41 μm, a fraction of low-Σ CSL boundaries of *F*_Σ_ = 64%, or of random boundaries *F*_R_ = 36%, and the fractal dimension of MRBC, *D*_R_ = 1.19. On the other hand, Type B specimen had the smaller average grain size of 17 μm, and the lower fraction of low Σ CSL boundaries of 44% (random boundaries of 56%) and the higher fractal dimension of MRBC of 1.63, in comparison with the Type A specimen.

From SEM observations of the surface of Type A specimen, it is evident that intergranular corrosion was inhibited by Σ3 CSL boundaries, but dominantly proceeded at random boundaries, as indicated by black arrows in Figure 14a. As a result, Type A specimen showed the higher resistance to intergranular corrosion, while Type B specimen was more heavily corroded because of the occurrence of a much higher fraction of random boundaries (*F*_R_ = 56%), resulting in the falling off of a number of grains and heavier roughening of the specimen surface, as seen from Figure 14c,d. These observations strongly suggest that the fractal dimension *D*_R_ can be a measure to predict the percolation potential for intergranular corrosion together with GBCD in SUS316L stainless steel.

In the case of nanocrystalline materials with grain size less than 100 nm, namely, the much higher density of grain boundaries, the more precise control of the grain boundary connectivity is required for controlling the percolation-dominating grain boundary phenomena. In view of this, a new approach to GBE based on the fractal analysis is expected to work for control of intrinsic or extrinsic intergranular brittleness caused by a dominant contribution of the network of percolation susceptive random boundaries. In particular, the MRBC first introduced by the present authors [87] is very likely applicable to solve the pending problem of poor ductility and intergranular brittleness, and further to confer desirable bulk mechanical properties to nanocrystalline materials through GBE.

**Figure 15:**
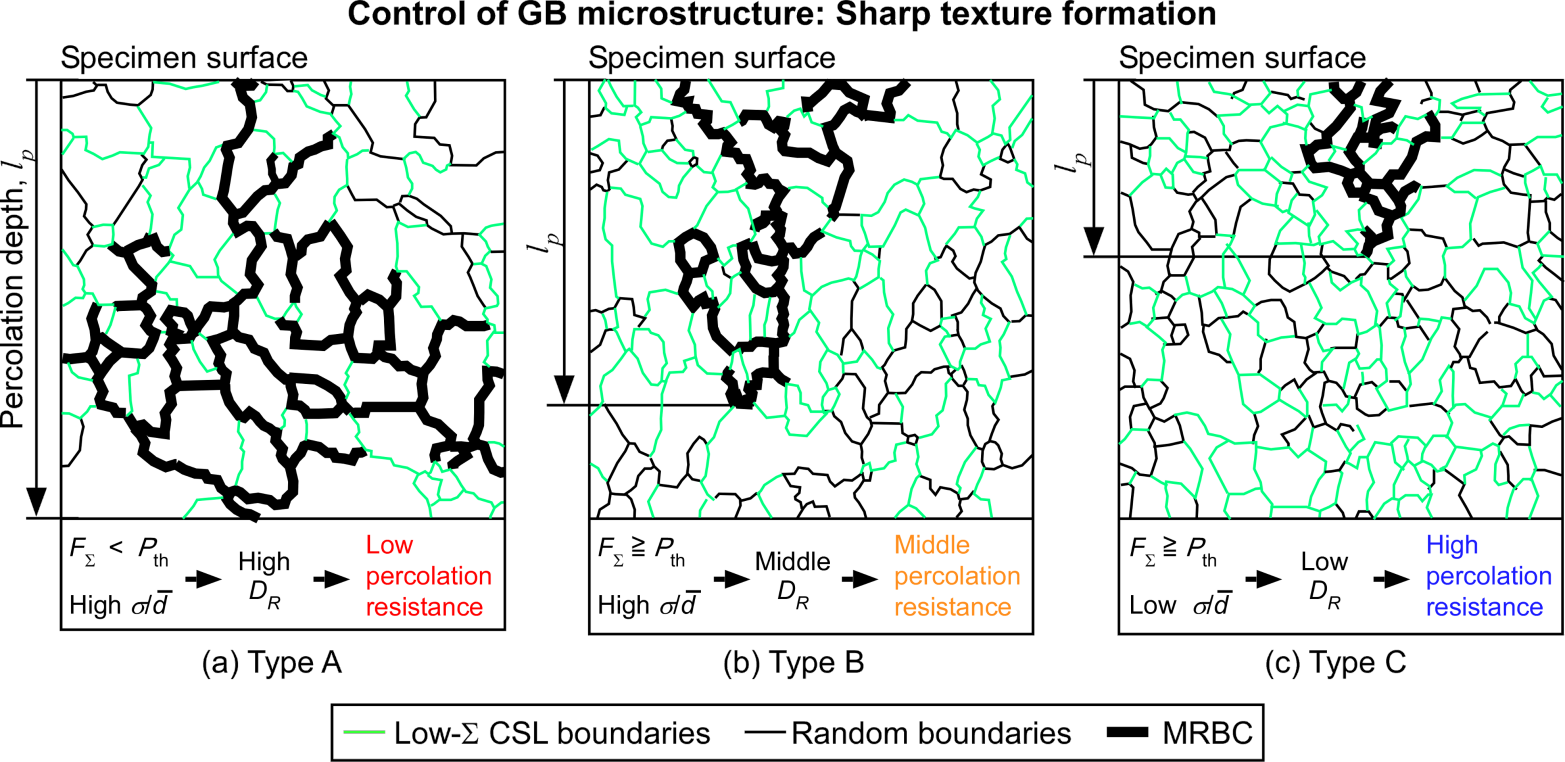
Schematic illustration showing the bulk mesoscopic propensity to percolation-related phenomena in the nanocrystalline materials with different grain boundary microstructures: (a) Type A with the highest value of *D*_R_, (b) Type B with the middle value of *D*_R_ and (c) Type C with the lowest value of *D*_R_. The process of grain boundary microstructure control is associated with the formation of a sharp texture, as observed in the electrodeposited nanocrystalline Ni and sputtered gold thin film specimens.

Figure 15 shows a schematic diagram illustrating our current knowledge of GBE through the incorporation of optimum grain boundary microstructure for desirable bulk properties controlled by the percolation-dominating intergranular phenomena in nanocrystalline materials. The illustrated grain boundary maps were drawn based on observed grain boundary maps to demonstrate real grain boundary microstructures with a sharp texture, in real nanocrystalline materials, such as electrodeposited Ni alloys and sputtered gold thin films mentioned later.

As a brief summary of our findings, first, Type A specimen may exhibit a low resistance to percolation-related intergranular degradation phenomena. This is because the fraction of low Σ CSL boundaries is lower than the percolation threshold value of MRBC and the high coefficient of variation of grain size distribution 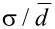, namely, the wide spread of grain size distribution. Second, since Type B specimen has the fractal dimension of MRBC being of the middle level of percolation resistance because of the fraction of low Σ CSL boundaries higher than the value of percolation threshold of MRBC and the high 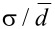. Third, Type C specimen, which has a high fraction of low-Σ CSL boundaries (more than the value of percolation threshold of MRBC and low 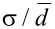, may bring about the highest percolation resistance, due to a low fractal dimension of MRBC.

### Electrical resistivity manipulated by GBE in nanocrystalline gold thin films

The improvement of electrical conductivity or precise control of electrical resistivity is required for the development of high performance electrical and magnetic materials for modern electronic devices such as MEMS and NEMS. It has been revealed that the electrical resistivity of individual grain boundaries strongly depends on the grain boundary character and structure from fundamental studies with orientation-controlled bicrystalline or coarse-grained metallic (Al) [123–124], semiconductor (Si) [125–126], and ceramic materials (ZnO) [127]. Nakamichi and Kino [124] have found from their systematic studies that low-energy/low-Σ CSL boundaries exhibit lower electrical resistivity than that of high-energy/random boundaries. Recent observations of electrical properties in silicon have revealed the dominant effects of the character of boundaries, their crystallographic plane, triple junctions and dopants. Accordingly, we expect that GBE based on the control of grain boundary microstructure must be useful for future improvement of electrical conductivity in polycrystalline materials, especially nanocrystalline materials which can be produced by advanced processing methods like various types of deposition methods and used in shape of tiny parts for microelectronic devices.

In thin film materials, the application of surface energy-driven grain growth is very useful and powerful for controlling the grain boundary microstructure. When a sharp texture is introduced in thin films with help of orientation-dependent surface free energy (namely by applying the surface energy-driven grain growth during annealing), specific low-Σ CSL boundaries can be preferentially introduced, depending on the type and the sharpness of texture [52,128–132]. In the case of gold thin films, the high fraction of low-Σ CSL boundaries predicted for the <111> rotation axis, such as Σ3, Σ7, Σ13, Σ19 and Σ21 boundaries preferentially occurred in relation to development of the sharp {111} texture, as observed in real polycrystalline materials [133–136].

**Figure 16:**
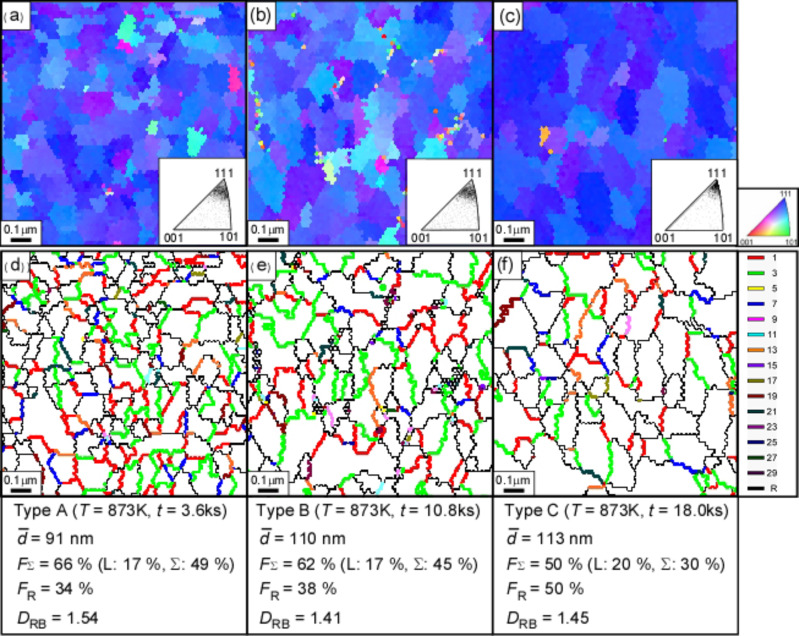
(a–c) OIM micrographs with inverse pole figures (IPF) of grain orientation distribution and (d–f) grain boundary microstructure for the gold thin film specimens sputtered on Pyrex grass substrates and subsequently annealed in Ar at 873 K for 3.6 ks (a,d), 10.8 ks (b,e) and 18.0 ks (c,f), respectively.

Figure 16 shows OIM micrographs and corresponding grain boundary maps for as-sputtered gold thin film specimen on Pyrex glass substrates and specimens annealed in Ar at 873 K for three different times (3.6, 10.8 and 18.0 ks). The three specimens subjected to different annealing times were designated as Type A, Type B and Type C specimens, respectively. The sharpness of the {111} texture was increased in these specimens by surface energy-driven grain growth during annealing. The Type A specimen had the smallest average grain size of 91 nm and the highest total fraction of low-angle (17%) and low-Σ CSL boundaries (49%), especially including CSL boundaries with Σ values predicted for <111> rotation axis (46%). The Type B specimen had an average grain size of 110 nm and has the high fraction of low-angle (17%) and low-Σ CSL boundaries (45%) similar to the Type A specimen. The Type C specimen had an average grain size (113 nm) similar to the Type B specimen, but the lowest total fraction of low-angle (20%) and low-Σ CSL boundaries (30%).

**Figure 17:**
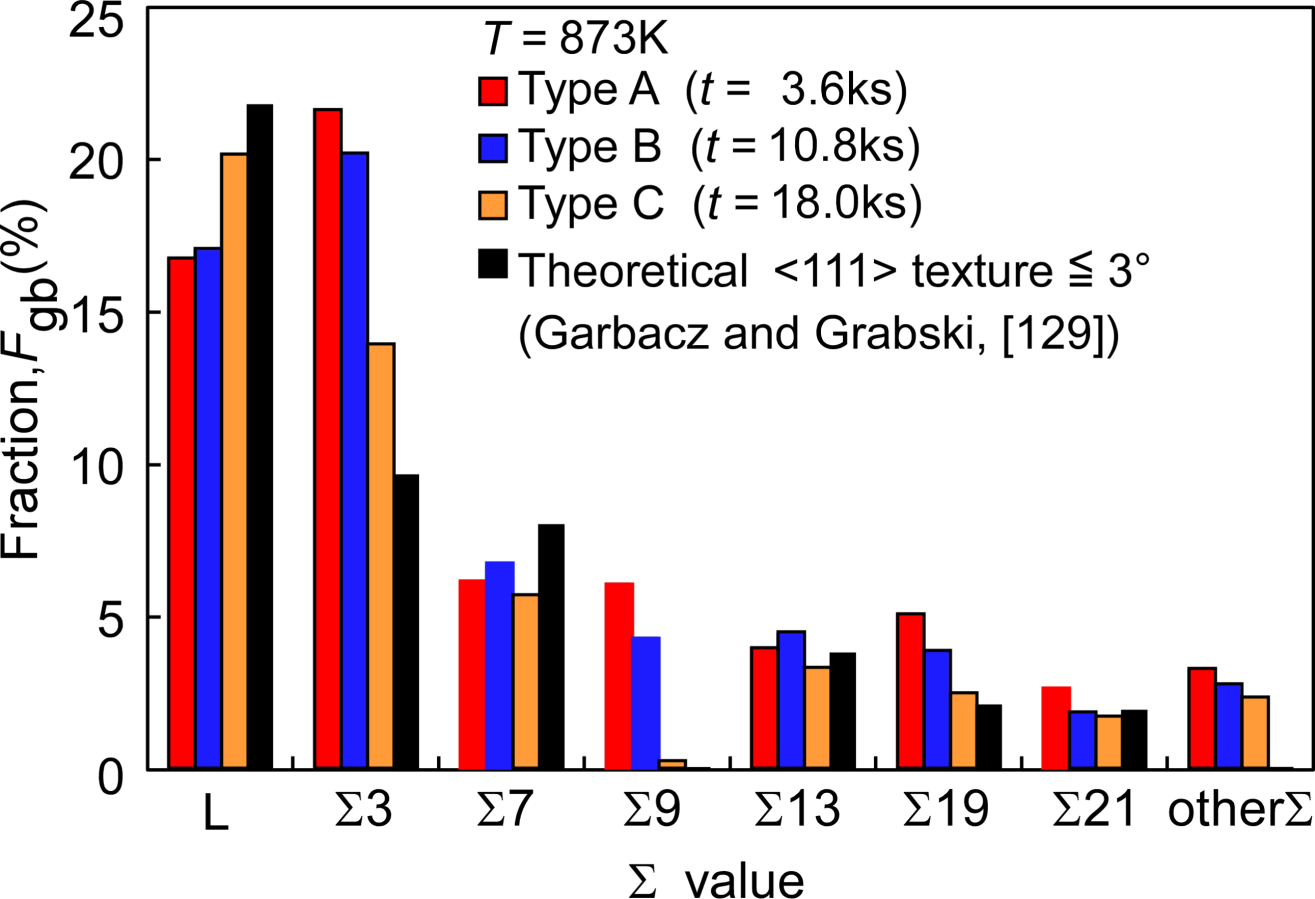
Change in grain boundary character distribution in the sputtered gold thin film specimens after annealing in Ar at 873 K for 3.6, 10.8 and 18.0 ks.

Figure 17 shows the results of the quantitative evaluation of GBCD obtained from the three differently processed gold thin film specimens, i.e., Type A, Type B and Type C. These specimens had a high fraction (17–20%) of low-angle boundaries, together with a high fraction low-Σ CSL boundaries with such specific Σ-values as Σ3, Σ7, Σ13, Σ19 and Σ21, predicted for the sharp <111> texture. Of particular interest is that Σ3, Σ13, Σ19 and Σ21 boundaries occurred with a much higher fraction than theoretical values of GBCD for the sharp {111} texture with deviation angle less than 3°. Moreover, a high fraction of Σ9 CSL boundaries also occurred in these specimens than in random polycrystals, although the predicated value for Σ9 is not available in the reported literature. This is probably because these specific low-Σ CSL boundaries with Σ3, Σ13, Σ19 and Σ21 belong to the group of CSL boundaries with the lowest grain boundary energy, and Σ9 CSL boundary belongs to the group of medium boundary energy in FCC materials [134,137].

**Figure 18:**
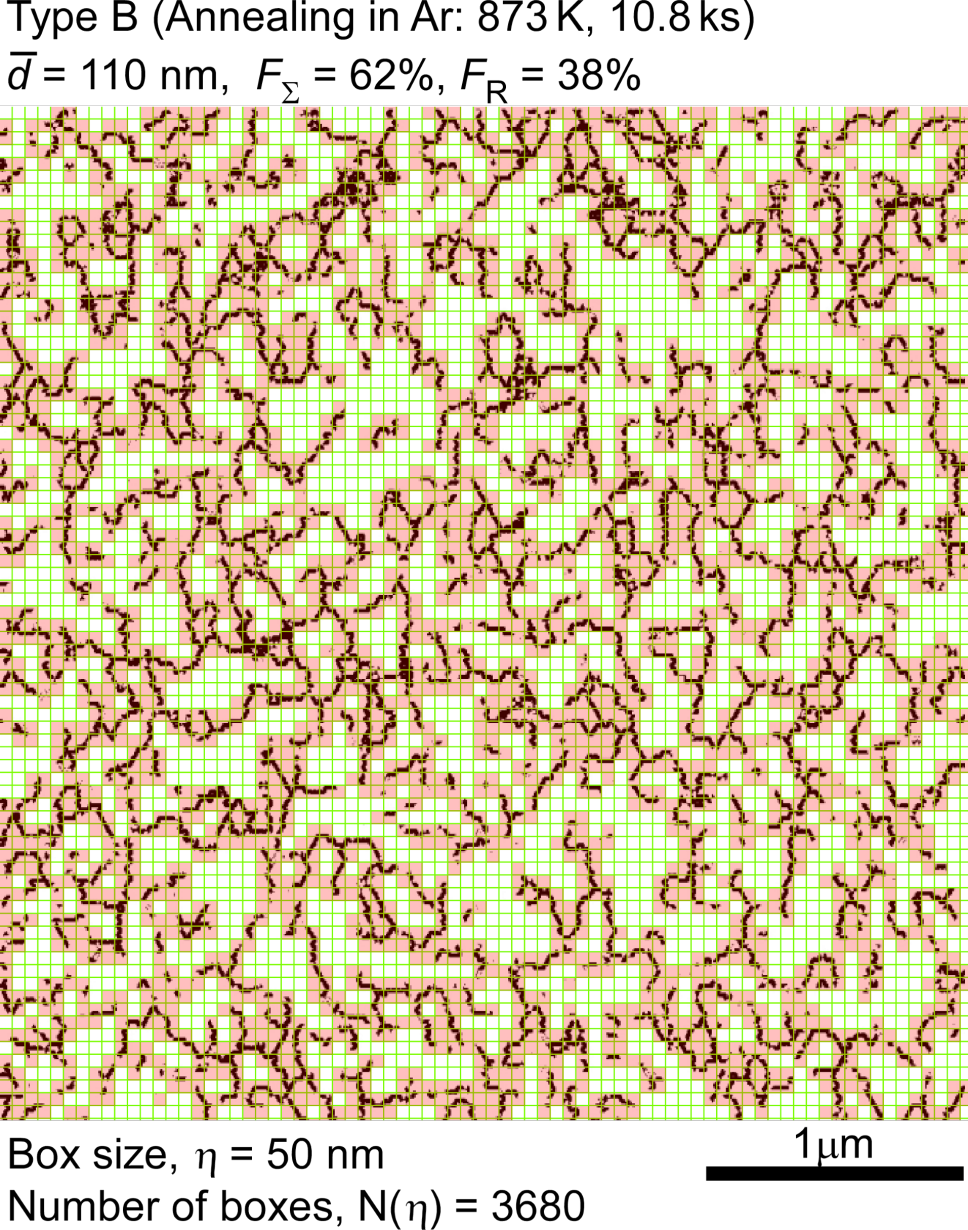
Example of the fractal analysis by box counting method for spatial distribution of random boundaries in gold thin film specimen (Type B).

In our new approach to GBE based on fractal analysis, we performed the fractal analysis for the grain boundary microstructures in the three different types of gold thin film specimens. One of the results obtained from our fractal analysis of GB microstructure in Type B specimen is shown in Figure 18, which indicates the result from the surface over a quite large area (≈3 × 3 μm), as being easily recognizable from the box size (50 nm) of the fractal analysis applied in this work. It is evident how interconnected random boundaries extend by depending on the grain boundary microstructures in individual studied gold thin film specimens, i.e., the heterogeneity of grain size distribution, GBCD, and grain boundary connectivity, or triple junction character distribution. To our knowledge, no literature is available which has reported such detailed information about grain boundary microstructure based on fractal analysis for real nanocrystalline materials, such as gold thin film in this work. From the image of the connectivity of random boundaries indicated by connected black lines in Figure 18, we can intuitively recognize and get such useful image of the grain boundary microstructure and quantitative evaluation of characteristic features from the combined analysis based on OIM and fractal analysis. This is yet to be achieved by computer simulation for understanding of grain boundary microstructure and bulk properties of polycrystalline and nanocrystalline materials.

Lastly, in order to give the reader a flavor of our new challenge of GBE for nanocrystalline materials introduced so far, let us just mention the most recent result on the effect of grain boundary microstructure on the electrical resistivity for the three types of gold thin film specimens with different grain boundary microstructure, already mentioned.

**Figure 19:**
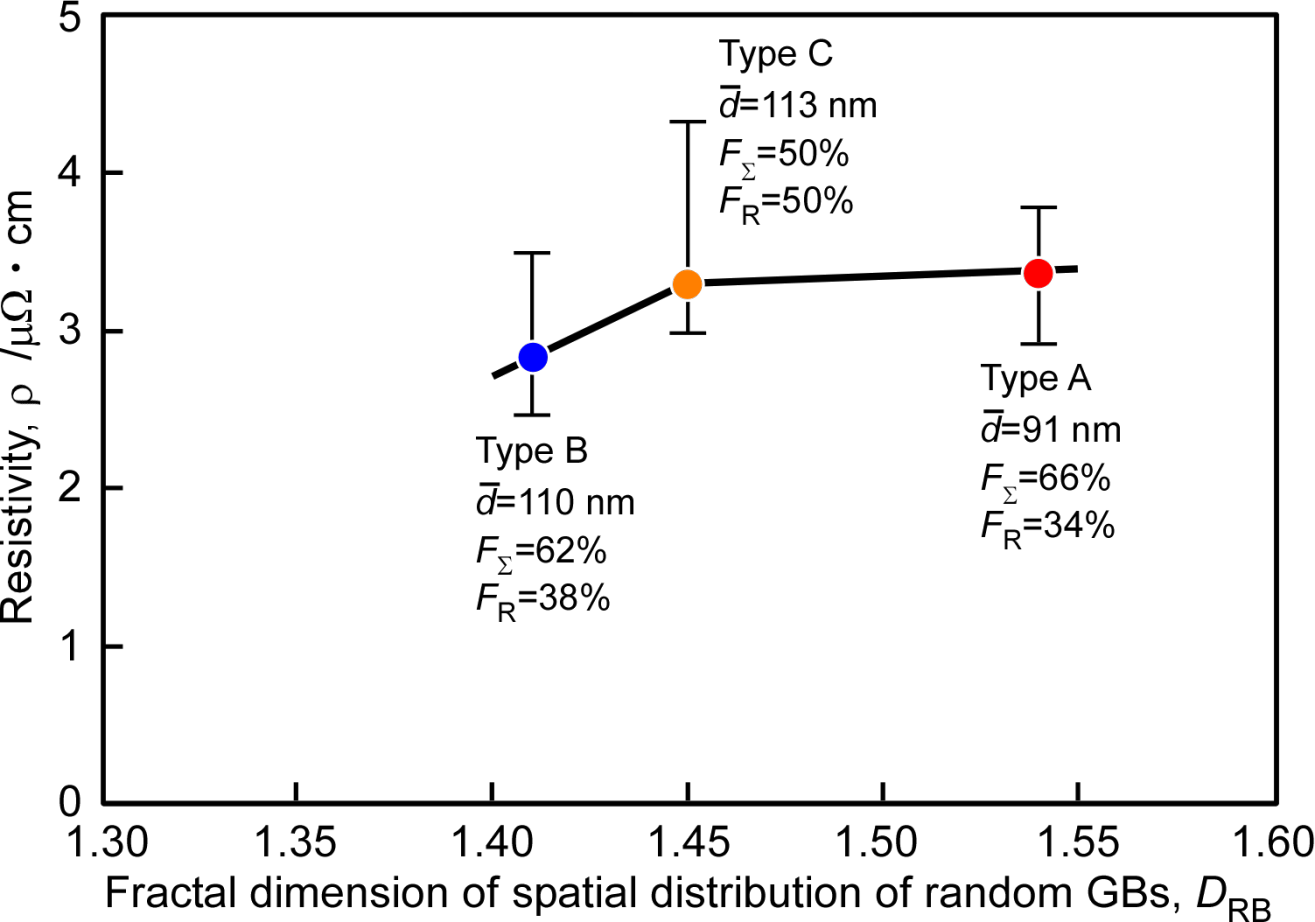
Relationship between the electrical resistivity and fractal dimension of spatial distribution of random boundaries in gold thin film specimens.

Figure 19 shows the experimental results on the electrical resistivity ρ as a function of the fractal dimension, associated with the spatial distribution of random boundaries as the primary scattering center of electrons in a polycrystal. Again it is evident that the electrical resistivity tends to increase systematically with increasing fractal dimension of random boundary connectivity. This result is first reported in this article to demonstrate the usefulness of our new approach to GBE for nanocrystalline functional materials. Here, a gold thin film as the most probable candidate as the highest performance electrical conductive material for advanced electronic devices.

Continuing the discussion about the results in Figure 19, the Type B specimen with a larger average grain size, namely due to a lower grain boundary density, showed lower electrical resistivity than the Type A specimen. It is feasible that the electrical resistivity in the latter with a higher grain boundary density became higher than that in the former even if a similar fraction of low-Σ CSL boundaries was kept in both specimens. The observation of the enhanced electrical resistivity in nanocrystalline materials was in good agreement with the previous works by Aus et al. [22]. Moreover, the electrical resistivity in the Type B specimen was lower than that in the Type C specimen. This result may provide some evidence that the electrical resistivity becomes lower in the specimen with a higher fraction of low-Σ CSL boundaries under the condition of similar GB density. It is natural that low-angle boundaries with a larger misorientation angle must have a higher value of the electrical resistivity owing to increase of the volume of dislocation core scattering at these grain boundaries. This is because the space of lattice dislocations becomes finer, decreasing with increasing misorientation angle [124]. We may conclude that a new approach to GBE based on the fractal analysis of GB microstructure is useful for precise control and improvement of electrical properties in nanocrystalline gold thin films. A historical background and a recent situation of GBE were introduced in the recent review by Watanabe [134].

## Conclusion

In this Review we have introduced our recent challenge, a new approach to grain boundary engineering (GBE) based on fractal analysis for grain boundary microstructures. This new approach has been undertaken quite recently for GBE in structural and functional polycrystalline materials, especially electrodeposited and sputtered nanocrystalline materials with an extremely high density of grain boundaries. It is shown that a more precise and quantitative evaluation of the effects of grain boundary microstructures are required for nanocrystalline materials than for ordinary polycrystalline materials. This is found by using the grain boundary character distribution (GBCD) and the grain boundary connectivity through SEM/EBSD/OIM analysis. It is well demonstrated that our new approach to GBE based on fractal analysis is very useful for the precise control of grain boundary microstructure-dependent bulk properties. This is especially helpful for mediating poor ductility and brittleness, which are long pending problems to be urgently solved in nanocrystalline materials. Our recent challenge of GBE based on fractural analysis for functional materials is also introduced. A solution was proposed for the future development of high performance functional materials, such as high performance electrical conductive materials, like gold thin films produced by the new approach of GBE.
